# Supplementary material for: Oxygen enhances antiviral innate immunity through maintenance of EGLN1-catalyzed proline hydroxylation of IRF3
Source: Nat Commun. 2024 Apr 26;15:3533. doi: 10.1038/s41467-024-47814-3 (PMC11053110; doi:10.1038/s41467-024-47814-3)

Supplementary Information

Oxygen enhances antiviral innate immunity through maintenance of EGLN1-catalysed proline hydroxylation of IRF3

Xing Liu[1,2, 3, 4] †, Jinhua Tang[1,5,] †, Zixuan Wang[1,3,] †, Chunchun Zhu[1, 3], Hongyan Deng[1], Xueyi Sun[1, 3], Guangqing Yu[1, 3], Fangjing Rong[1,3], Xiaoyun Chen[1, 3], Qian Liao[1, 3], Shuke Jia[1, 3], Wen Liu[1, 3], Huangyuan Zha[1], Sijia Fan[1], Xiaolian Cai[1], Jian-Fang Gui[1, 2, 3, 4] and Wuhan Xiao[1, 2, 3, 4,] *

[1] Key Laboratory of Breeding Biotechnology and Sustainable Aquaculture, Institute of Hydrobiology, Chinese Academy of Sciences, Wuhan, P. R. China; [2] Hubei Hongshan Laboratory, Wuhan, 430070, P. R. China; [3] University of Chinese Academy of Sciences; Beijing, P. R. China; [4] The Innovation Academy of Seed Design, Chinese Academy of Sciences; Wuhan, P. R. China; [5] Department of Pharmacy, Women and Children's Hospital of Chongqing Medical University; Chongqing, 401147, P. R. China.

*Correspondence and requests for materials should be addressed to W. X. (email: w-xiao@ihb.ac.cn)

Supplementary Figure 1-17

Supplementary Table 1, Table 2

Description of Additional Supplementary Information

Supplementary Fig. 1. Hypoxia suppresses antiviral gene expression in response to viral infection independently of HIF signaling. Related to Fig. 1.

Supplementary Fig. 2. *EGLN1* positively regulates cellular antiviral immune responses. Related to Fig. 2.

Supplementary Fig. 3. *EGLN1,* but not *EGLN2* and *EGLN3,* positively regulates cellular antiviral responses. Related to Fig. 2.

Supplementary Fig. 4. Evaluation of the specific inhibitor of EGLN prolyl hydroxylase, FG4592 (Roxadustat). Related to Fig. 2.

Supplementary Fig. 5. Transcriptome analysis of *Egln1*$^{+/+}$ and *Egln1*$^{-/-}$ MEF cells in response to VSV infection. Related to Fig. 2.

Supplementary Fig. 6. Disruption of *Egln1* in mice results in increased susceptibility to lethal viral infection. Related to Fig. 3.

Supplementary Fig. 7. Disruption of *egln1* in zebrafish results in increased susceptibility to lethal viral infection. Related to Fig. 4.

Supplementary Fig. 8. Inhibition of egln1 activity in zebrafish by FG4592 results in increased susceptibility to lethal viral infection. Related to Fig. 4.

Supplementary Fig. 9. EGLN1 interacts with IRF3. Related to Fig. 5.

Supplementary Fig. 10. *EGLN1* promotes cellular antiviral immune response dependent on IRF3 and type I IFN. Related to Fig. 5.

Supplementary Fig. 11. EGLN1 hydroxylates IRF3 at proline 10. Related to Fig. 6.

Supplementary Fig. 12. EGLN1 enhances IRF3 phosphorylation and dimerization. Related to Fig. 7.

Supplementary Fig. 13. Hydroxylation of IRF3 at proline 10 enhances IRF3 activation in cellular antiviral immune responses. Related to Fig. 8.

Supplementary Fig. 14. Generation of Irf3_P10A mutant mice. Related to Fig. 9.

Supplementary Fig. 15. Irf3 prolyl hydroxylation deficiency attenuates antiviral innate immunity in mice. Related to Fig. 9.

Supplementary Fig. 16. Detection of HIF1α protein in wild-type and Irf3_P10A mutant mice after virus infection. Related to Fig. 9.

Supplementary Fig. 17. A working model of oxygen in antiviral innate immunity.

Supplementary Table 1. Reagents.

Supplementary Table 2. The primer sequences.

Original Western blots in Supplementary Figures

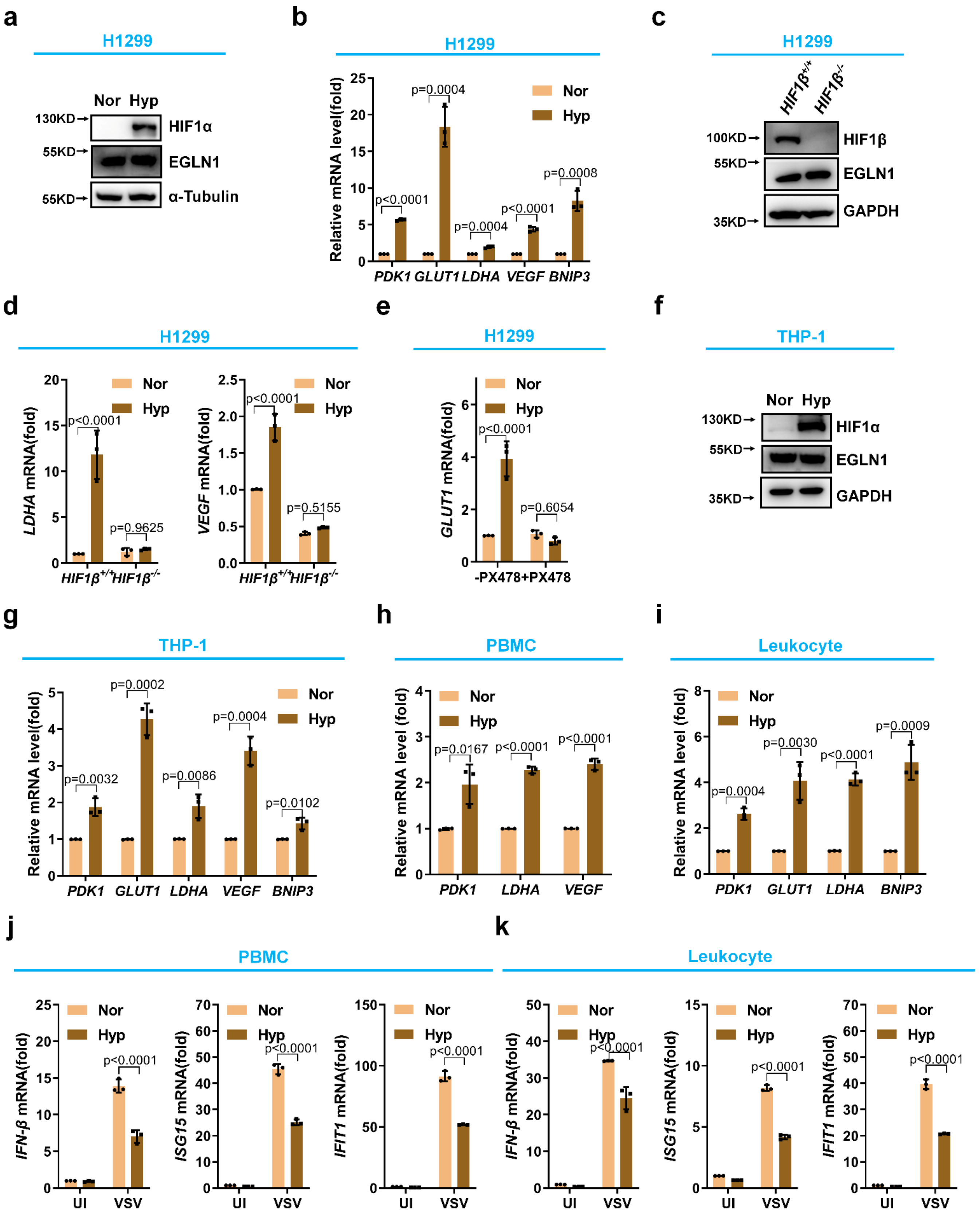

Supplementary Fig. 1. Hypoxia suppresses antiviral gene expression in response to viral infection independently of HIF signaling. Related to Fig. 1.

(a) Immunoblotting (IB) of HIF1α protein in H1299 cells under normoxia (21% O_2) or hypoxia (1% O_2). (b) Quantitative real-time PCR (qPCR) analysis of *PDK1*, *GLUT1*, *LDHA*, *VEGF* and *BNIP3* mRNA in H1299 cells

under normoxia (21% O_2) or hypoxia (1% O_2) for 8 hours. (c) Validation of HIF1β protein in *HIF1β*$^{+/+}$ and *HIF1β* $^{-/-}$ H1299 cells. (d) qPCR analysis of *LDHA* and *VEGF* mRNA in *HIF1β*$^{+/+}$ and *HIF1β* $^{-/-}$ H1299 cells under normoxia (21% O_2) or hypoxia (1% O_2) for 8 h. (e) qPCR analysis of *GLUT1* mRNA in H1299 cells treated with or without PX478 (10 μM) and cultured under normoxia (21% O_2) or hypoxia (1% O_2) for 8 h. (f) Immunoblotting of HIF1α and EGLN1 proteins in THP-1 cells under normoxia (21% O_2) or hypoxia (1% O_2). (g) qPCR analysis of *PDK1, GLUT1, LDHA*, *VEGF* and *BNIP3* mRNA in THP-1 cells under normoxia (21% O_2) or hypoxia (1% O_2) for 8 h. (h) qPCR analysis of *PDK1, LDHA,* and *VEGF* mRNA in PBMC cells under normoxia (21% O_2) or hypoxia (1% O_2) for 8 h. (i) qPCR analysis of *PDK1*, *GLUT1*, *LDHA*, and *BNIP3* mRNA in leukocyte cells under normoxia (21% O_2) or hypoxia (1% O_2) for 8 h. (j) qPCR analysis of *IFN-β, ISG15,* and *IFIT1* mRNA in PBMC cells under normoxia (21% O_2) or hypoxia (1% O_2) for 8h, infected without (UI) or with VSV for 8 h. (k) qPCR analysis of *IFN-β, ISG15,* and *IFIT1* mRNA in leukocyte cells under normoxia (21% O_2) or hypoxia (1% O_2) for 12 h, infected without (UI) or with VSV for 8 h. Data in (**a, c, f**) are representative from three independent experiments. Data in (**b, g, h, i**) are presented as mean ± S.D., two-tailed student's *t* test; *n*=3 biological independent experiments. Data in (**d, e, j, k**) are presented as mean ± S.D., two-way ANOVA; *n*=3 biological independent experiments. Source data are provided as a Source Data file.

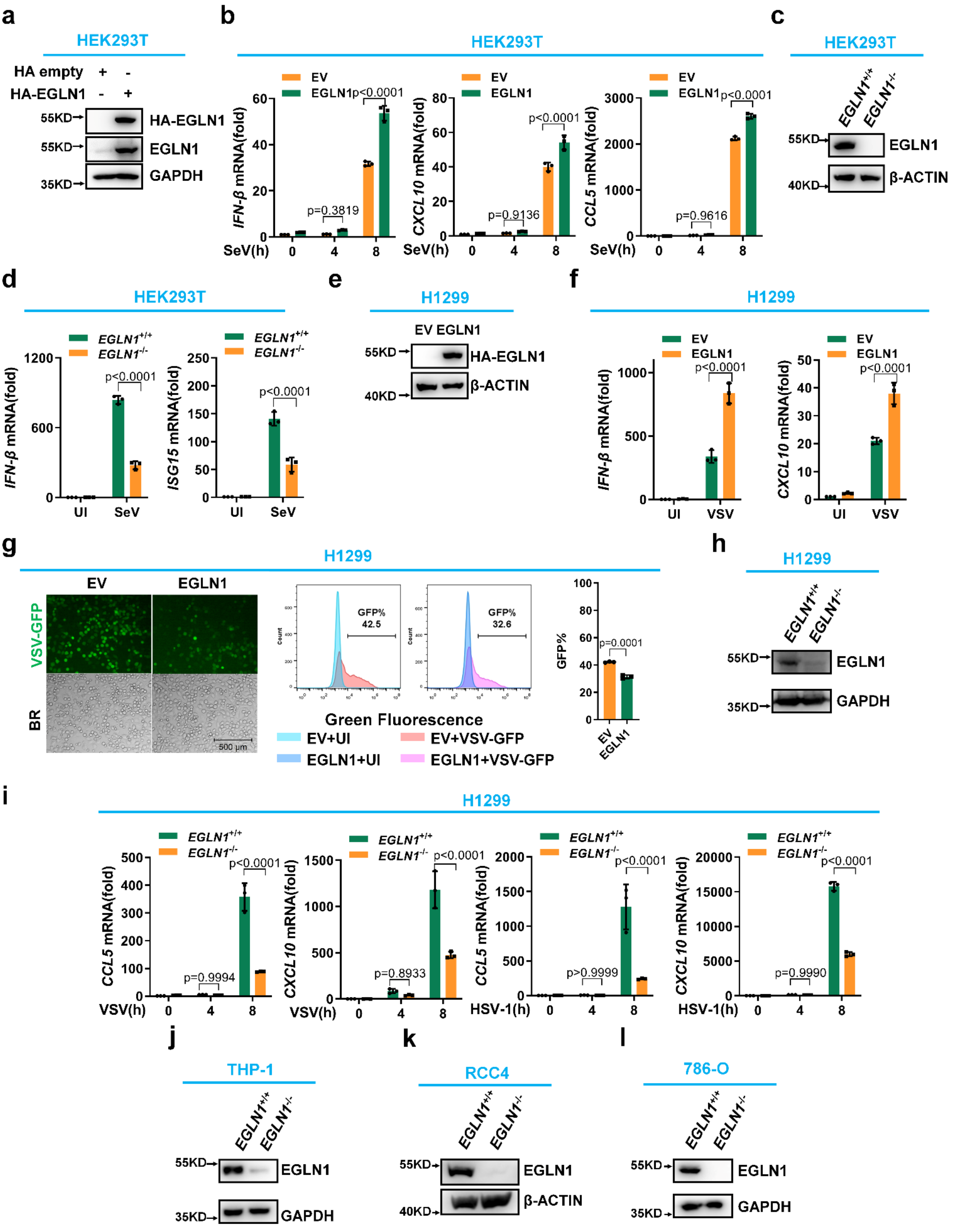

Supplementary Fig. 2. *EGLN1* positively regulates cellular antiviral immune responses. Related to Fig. 2.
(a) Validation of EGLN1 protein in HEK293T cells transfected with the HA empty vector or the plasmid expressing HA–*EGLN1*. (b) qPCR of *IFN-β*, *CXCL10*, and *CCL5* mRNA in HEK293T cells transfected with the HA empty

vector (EV) or the plasmid expressing HA–*EGLN1* for 24 h, followed by infection with SeV for 0, 4 and 8 h. (c) Validation of EGLN1 protein in *EGLN1* $^{+/+}$ or *EGLN1* $^{-/-}$ HEK293T cells. (d) qPCR analysis of *IFN-β* and *ISG15* mRNA in *EGLN1* $^{+/+}$ or *EGLN1* $^{--/-}$ HEK293T cells infected without (UI) or with SeV for 8 h. (e) Validation of overexpressed EGLN1 in H1299 cells. (f) qPCR analysis of *IFN-β* and *CXCL10* mRNA in H1299 cells transfected with the HA empty vector (EV) or the plasmid expressing HA-*EGLN1* for 24 h, followed by uninfected (UI) or infected with VSV for 8 h. (g) H1299 cells were transfected with the HA empty vector (EV) or the plasmid expressing HA-*EGLN1* for 24 h, followed by infection without (UI) or with VSV-GFP virus for 12 h, and viral infectivity was detected by fluorescence microscopy or flow cytometry analysis. (h) Validation of EGLN1 protein in *EGLN1* $^{+/+}$ and *EGLN1* $^{-/-}$ H1299 cells. (i) qPCR analysis of *CCL5* and *CXCL10* mRNA in *EGLN1*$^{+/+}$ and *EGLN1*$^{-/-}$ H1299 cells infected with VSV (left two panels) or HSV-1 (right two panels) for 0, 4, and 8 h. (j) Validation of EGLN1 protein in *EGLN1* $^{+/+}$ and *EGLN1* $^{-/-}$ THP-1 cells. (k) Validation of EGLN1 protein in *EGLN1* $^{+/+}$ and *EGLN1* $^{-/-}$ RCC4 cells. (l) Validation of EGLN1 protein in *EGLN1* $^{+/+}$ and *EGLN1* $^{-/-}$ 786-O cells. UI, uninfected; EV, empty vector; BR, bright field. Data in (**a, c, e, g, h, j-l**) are representative from three independent experiments. Data in (**b, d, f, i**) are presented as mean ± S.D., two-way ANOVA; *n*=3 biological independent experiments. Data in (**g**) are presented as mean ± S.D., two-tailed student's *t* test; *n*=3 biological independent experiments. Source data are provided as a Source Data file.

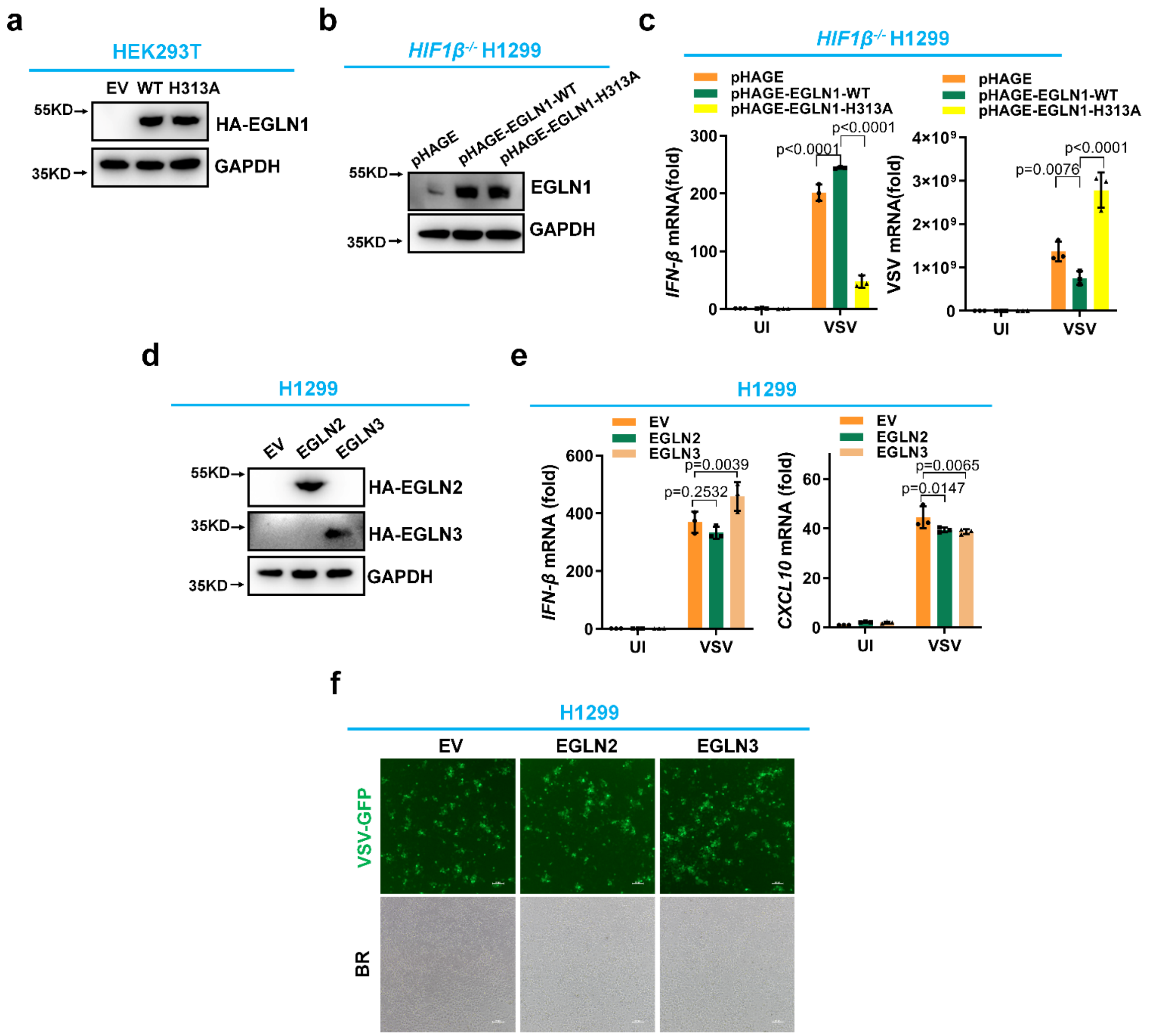

Supplementary Fig. 3. *EGLN1*, but not *EGLN2* and *EGLN3*, significantly enhances cellular antiviral responses. Related to Fig. 2.

(a) Validation of overexpressed HA-EGLN1 (WT) or HA-EGLN1-H313A (H313A) in HEK293T cells. (b) Validation of wild-type EGLN1 (WT) or the enzymatically inactive mutant of EGLN1 (H313A) in *HIF1β* $^{-/-}$ H1299 cells infected with lentivirus. (c) qPCR analysis of *IFN-β* mRNA or VSV virus mRNA in *HIF1β* $^{-/-}$ H1299 cells infected with lentivirus expressing empty vector (EV), wild-type EGLN1 (EGLN1-WT) or the catalytically inactive mutant of EGLN1 (EGLN1-H313A), followed by infection without (UI) or with VSV for 8 h. (d) Validation of overexpressed EGLN2 and EGLN3 in H1299 cells by immunoblotting with anti-HA antibody. (e) qPCR analysis of *IFN-β* and *CXCL10* mRNA in H1299 cells transfected with the HA empty vector (EV), the plasmid expressing HA-*EGLN2*, or the plasmid expressing HA-*EGLN3*, followed by infection without (UI) or with VSV for 8 h. (f) H1299 cells were transfected with the HA empty vector (EV), the plasmid expressing HA-*EGLN2*, or the plasmid expressing HA-*EGLN3*, followed by infection with VSV-GFP viruses for 12 h, and viral infectivity was detected by fluorescence microscopy. UI, uninfected; EV, empty vector; BR, bright field. Data in (**a, b, d, f**) are representative from three independent experiments. Data in (**c, e**) are presented as mean ± S.D., two-way ANOVA; *n*=3 biological independent experiments. Source data are provided as a Source Data file.

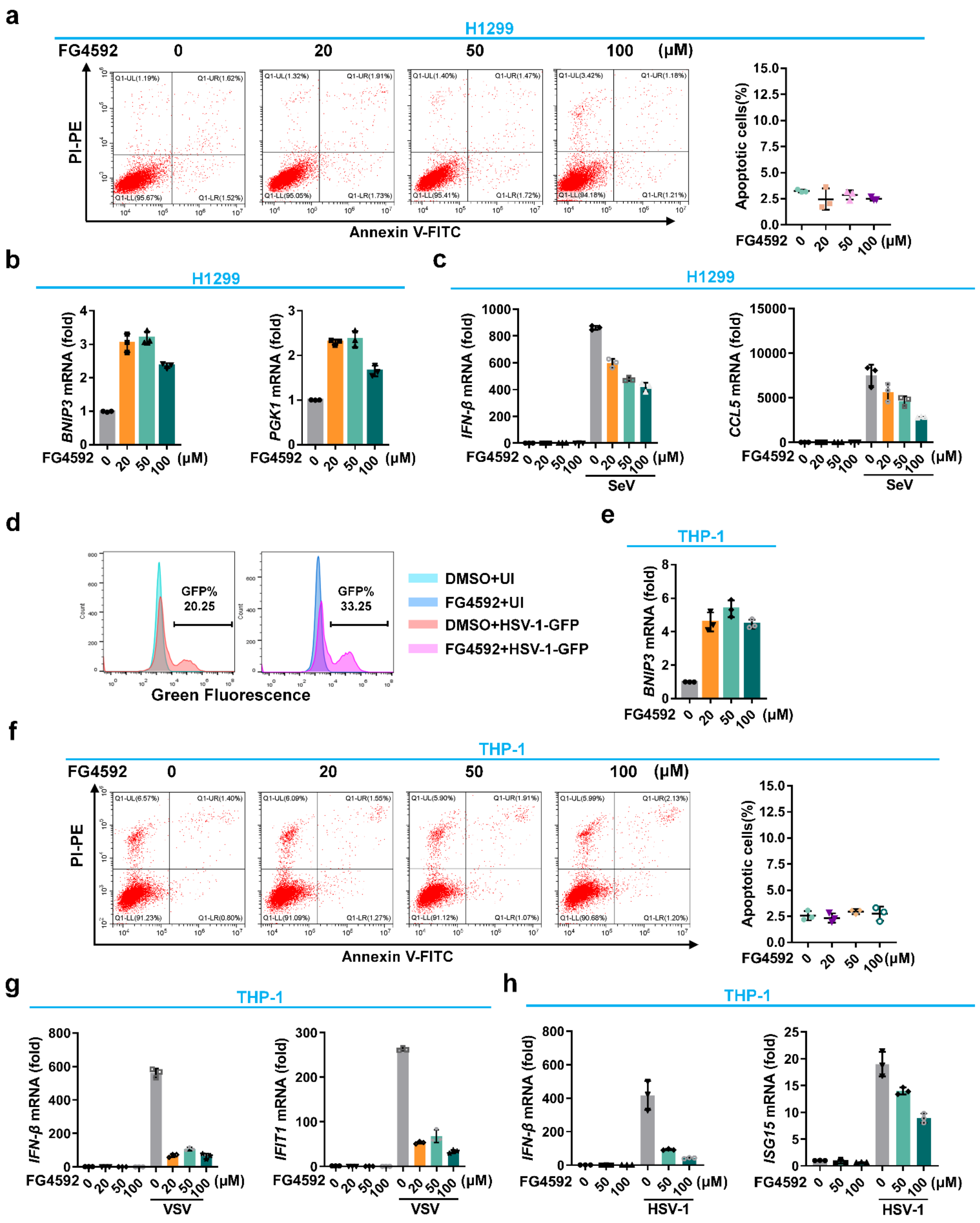

Supplementary Fig. 4. Evaluation of the specific inhibitor of EGLN prolyl hydroxylase, FG4592 (Roxadustat). Related to Fig. 2.

(a) H1299 cells were treated with FG4592 (from 0 to 100 μM) for 24 h and cell apoptosis was determined by staining with PI and Annexin V; quantitation of apoptotic cells is shown in the right panel. (b) qPCR analysis of *BNIP3* and *PGK1* mRNA in H1299 cells treated with FG4592 (from 0 to 100 μM) for 24 h. (c) qPCR analysis of *IFN-β* and *CCL5* mRNA in H1299 cells treated with FG4592 (from 0 to 100 μM) for 6 h, followed by infection without (UI) or with SeV for 8 h. (d) H1299 cells were treated with DMSO (vehicle control) or FG4592 (20 μM) for 6 h, followed by infection without (UI) or with VSV-GFP virus for 12h, and viral infectivity was detected by flow cytometry analysis. (e) qPCR analysis of *BNIP3* mRNA in THP-1 cells treated with FG4592 (from 0 to 100 μM) for 6 h. (f) THP-1 cells were treated with FG4592 (from 0 to 100 μM) for 24 h and cell apoptosis was determined by staining with PI and Annexin V; quantitation of apoptotic cells is shown in the right panel. (g) qPCR analysis of *IFN-β* and *IFIT1* mRNA in THP-1 cells treated with FG4592 (from 0 to 100 μM) for 6 h, followed by infection without (UI) or with VSV for 8 h. (h) qPCR analysis of *IFN-β* and *ISG15* mRNA in THP-1 cells treated with FG4592 (from 0 to 100 μM) for 24 h, followed by infection without or with HSV-1 for 8 h. Data in (**a, d, f**) are representative from three independent experiments. Data in (**a-c, e-h**) are presented as mean ± S.D.; *n*=3 biological independent experiments. Source data are provided as a Source Data file.

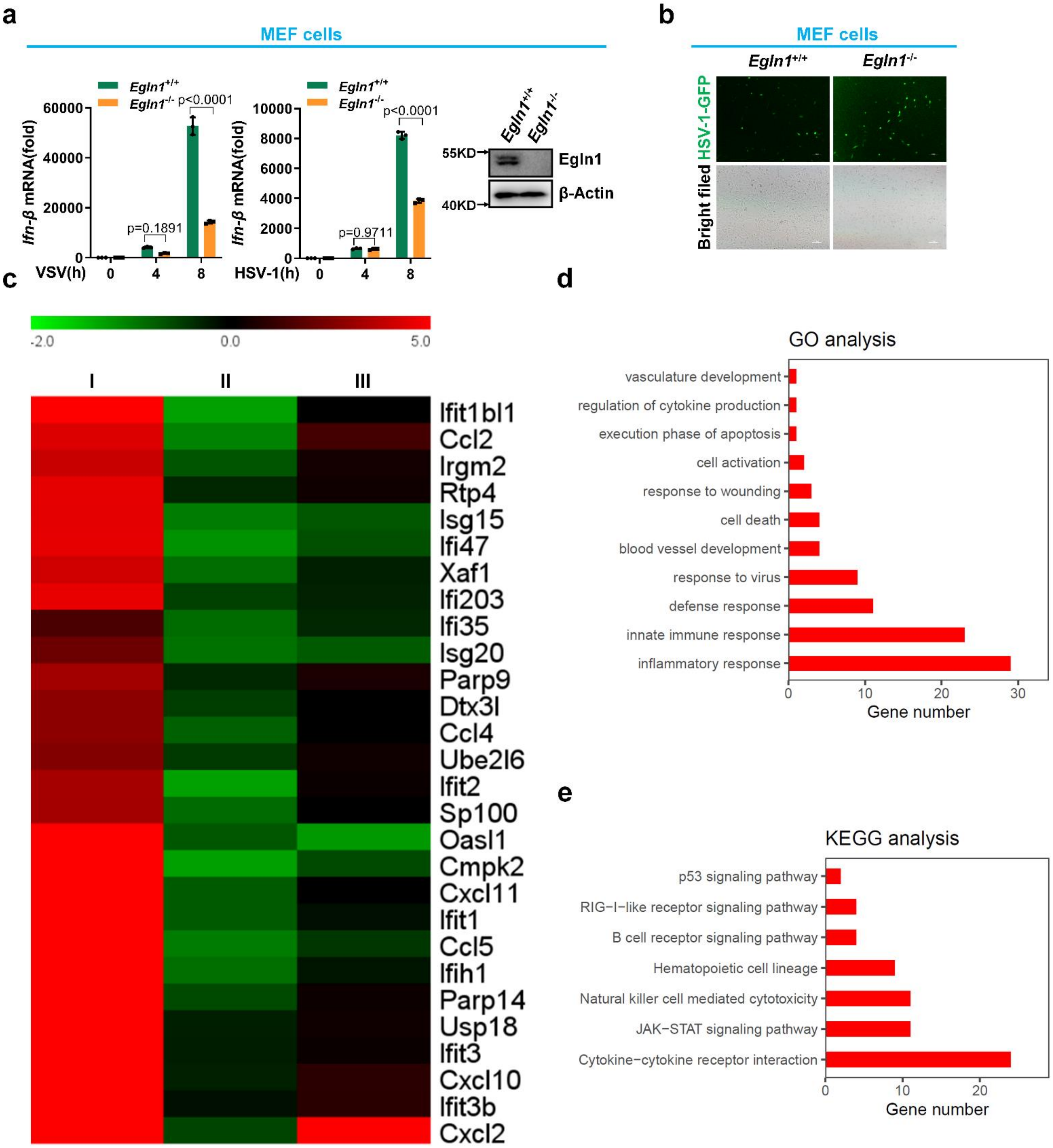

Supplementary Fig. 5. Transcriptome analysis of *Egln1*$^{+/+}$ and *Egln1*$^{-/-}$ MEF cells in response to VSV infection. Related to Fig. 2.

(a) qPCR analysis of *Ifn-β* mRNA in *Egln1*$^{+/+}$ and *Egln1*$^{-/-}$ MEF cells infected with VSV or HSV-1 for 0, 4 and 8 h. (b) *Egln* $^{+/+}$ and *Egln1* $^{-/-}$ MEF cells were infected with HSV-1-GFP virus for 12 h, and viral infectivity was detected by fluorescence microscopy. (c) Heatmap for selected Interferon-stimulated genes (ISGs) in *Egln1*$^{+/+}$ and *Egln1*$^{-/-}$ MEF cells uninfected (-) or infected (+) with VSV for 8 h. Expression fold changes between two samples were Log2 transformed, and these values were used to generate the heatmap of the indicated ISGs using MeV software. I: VSV-infected *Egln1*$^{+/+}$/*Egln1*$^{+/+}$; II: VSV-infected *Egln1*$^{-/-}$/ VSV-infected *Egln1*$^{+/+}$; III: *Egln1*$^{-/-}$ / *Egln1*$^{+/+}$. (d) Gene Ontology

(GO) enrichment analysis for the DEGs was performed using Cluster Profiler version 3.8. for the activated genes in *Egln1*$^{-/-}$ MEF cells and *Egln1*$^{+/+}$ MEF cells infected with VSV for 8 h. (e) Kyoto Encyclopedia of Genes and Genomes (KEGG) enrichment analyses for the DEGs were performed using Cluster Profiler version 3.8. for the activated genes in *Egln1*$^{-/-}$ MEF cells and *Egln1*$^{+/+}$ MEF cells infected with VSV for 8 h. Data in (**a, b**) are representative from three independent experiments. Data in (**a**) are presented as mean ± S.D., two-way ANOVA; *n*=3 biological independent experiments. Source data are provided as a Source Data file or uploaded to the GEO Datasets.

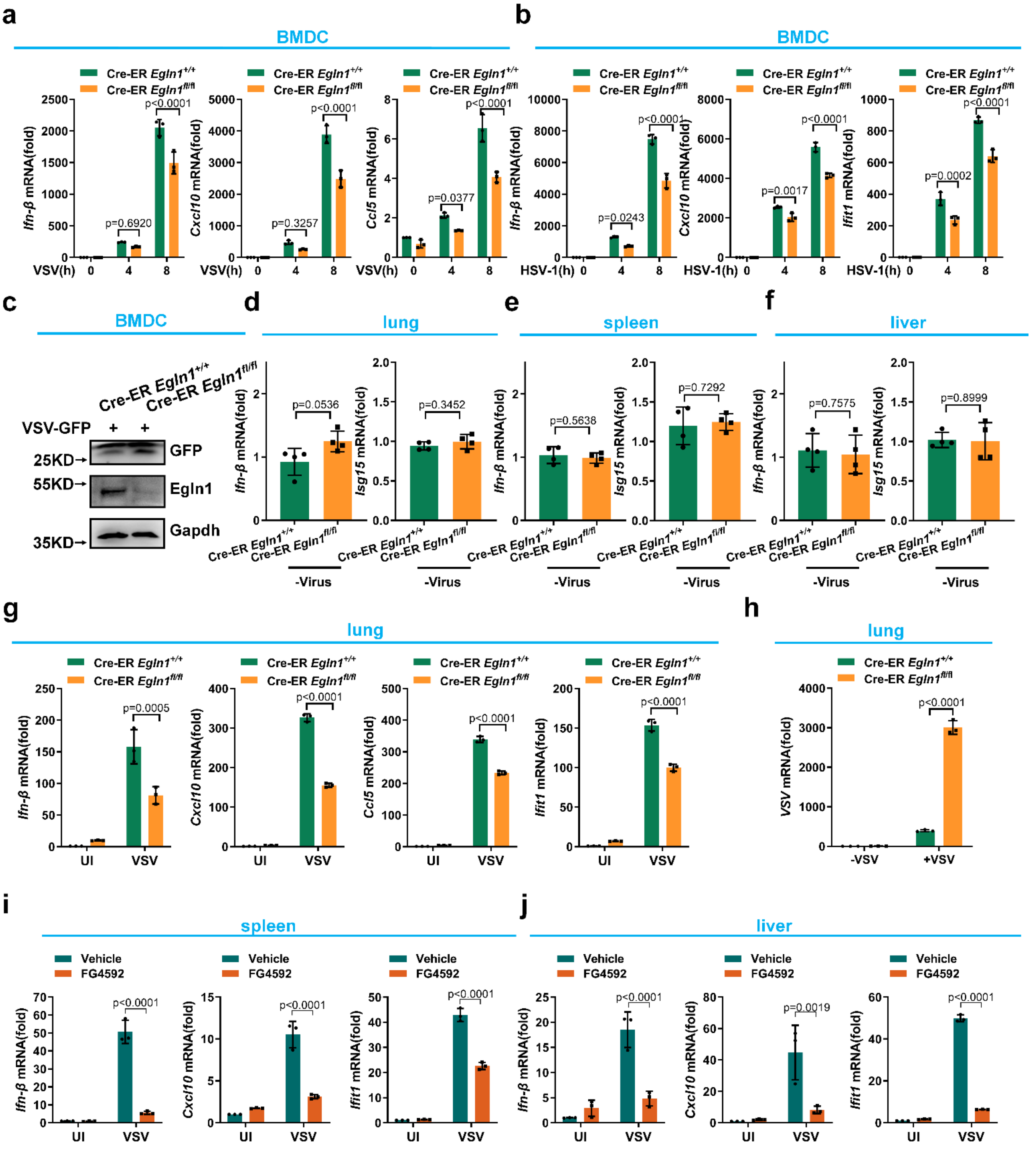

Supplementary Fig. 6. Disruption of *Egln1* in mice results in increased susceptibility to lethal viral infection. Related to Fig. 3.

(a) qPCR analysis of *Ifn-β, Cxcl10* and *Ccl5* mRNA in Cre-ER *Egln1*$^{+/+}$ and Cre-ER *Egln1*$^{fl/fl}$ BMDC cells infected with VSV for 0, 4 and 8 h. (b) qPCR analysis of *Ifn-β, Cxcl10* and *Ifit1* mRNA in Cre-ER *Egln1*$^{+/+}$ and Cre-ER *Egln1*$^{fl/fl}$ BMDC cells infected with HSV-1 for 0, 4 and 8 h. (c) Cre-ER *Egln1*$^{+/+}$ and Cre-ER *Egln1*$^{fl/fl}$ BMDC cells were infected with VSV-GFP virus for 12 h, and viral infectivity was detected by immunoblotting with anti-GFP antibody. (d-f) qPCR analysis of *Ifn-β* and *Isg15* mRNA in lung (d), spleen (e), and liver (f) of tamoxifen-treated Cre-ER *Egln1*$^{+/+}$ and Cre-ER *Egln1*$^{fl/fl}$ mice. (g) qPCR analysis of *Ifn-β, Cxcl10, Ccl5* and *Ifit1* mRNA in lung of

tamoxifen-treated Cre-ER *Egln1*$^{+/+}$ and Cre-ER *Egln1*$^{fl/fl}$ mice infected with VSV (1×10^7 PFU per mouse) for 24 h. (h) qPCR analysis of VSV mRNA in lung of tamoxifen-treated Cre-ER *Egln1*$^{+/+}$ and Cre-ER *Egln1*$^{fl/fl}$ mice infected with VSV (1×10^7 PFU per mouse) for 24 h. (i, j) qPCR analysis of *Ifn-β, Cxcl10*, and *Ifit*1 mRNA in spleen (i) and liver (j) of mice treated with vehicle control or FG4592 (10 mg/kg) and injected intraperitoneally with VSV (1×10^7 PFU per mouse) for 24 h. Data in (**a, b, g-j**) are presented as mean ± S.D., two-way ANOVA; *n*=3 biological independent experiments. Data in (**c**) are representative from three independent experiments. Data in (**d-f**) are presented as mean ± S.D., two-tailed student's *t* test; *n*=3 biological independent experiments. Source data are provided as a Source Data file.

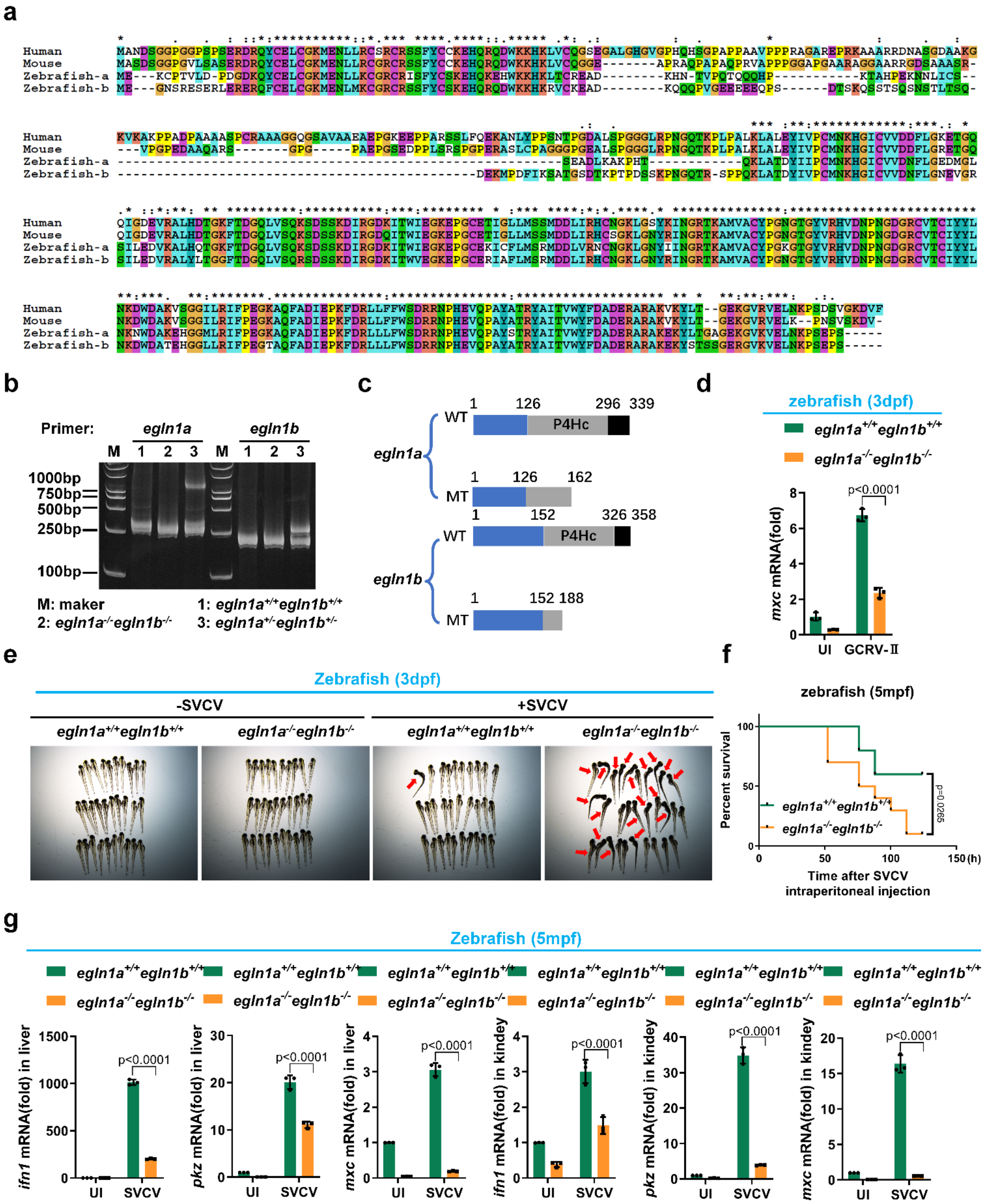

Supplementary Fig. 7. Disruption of *egln1* in zebrafish results in increased susceptibility to lethal viral infection. Related to Fig. 4.

(a) Alignment of EGLN1 amino acid sequences from human, mouse, and zebrafish EGLN1 amino acid sequences. (b) Genomic DNA was prepared from the indicated lines (*egln1a*$^{+/+}$*egln1b*$^{+/+}$, *egln1a*$^{-/-}$*egln1b*$^{-/-}$ and *egln1*$^{+/-}$*egln1b*$^{+/-}$), and

zebrafish *egln1a* and *egln1b* disruption was verified by the heteroduplex mobility assay (HMA). (c) The resulting protein information in *egln1a* or *egln1b* null zebrafish. (d) qPCR analysis of *mxc* mRNA in wild-type (*egln1a*$^{+/+}$*egln1b*$^{+/+}$) and *egln1a* and *egln1b* double knockout (*egln1a*$^{-/-}$*egln1b*$^{-/-}$) zebrafish larvae (3 dpf, n=30) infected without (UI) or with GCRV-II for 18 h. (e) Representative images of wild-type (*egln1a*$^{+/+}$*egln1b*$^{+/+}$) and *egln1a* and *egln1b* double knockout (*egln1a*$^{-/-}$*egln1b*$^{-/-}$) zebrafish larvae (3 dpf, n=30) infected without (-) or with (+) SVCV for 24 h. The dead larvae showed lack of movement, curved body, and a body degeneration as indicated by the red arrows. (f) Survival (Kaplan-Meier curve) of adult wild-type (*egln1a*$^{+/+}$*egln1b*$^{+/+}$) and *egln1a* & *egln1b*-double knockout (*egln1a*$^{-/-}$*egln1b*$^{-/-}$) zebrafish (5 mpf) injected intraperitoneally with SVCV (~6 ×10^7 $TCID_{50}$/ml) and monitored for 150 h. Statistical analysis was performed using the log-rank test (n=10 for each group). (g) qPCR analysis of *ifn1*, *pkz* and *mxc* mRNA in liver (left three panels) and kidney (right three panels) of adult wild-type (*egln1a*$^{+/+}$*egln1b*$^{+/+}$) and *egln1a* & *egln1b* double knockout (*egln1a*$^{-/-}$*egln1b*$^{-/-}$) zebrafish (5 months post-fertilization; 5 mpf) injected intraperitoneally without (UI) or with SVCV (~6 ×10^7 $TCID_{50}$/ml) for 24 h. UI, uninfected. Data in (**d, g**) are presented as mean ± S.D., two-way ANOVA; *n*=3 biological independent experiments. Data in (**e**) are representative from three independent experiments. Source data are provided as a Source Data file.

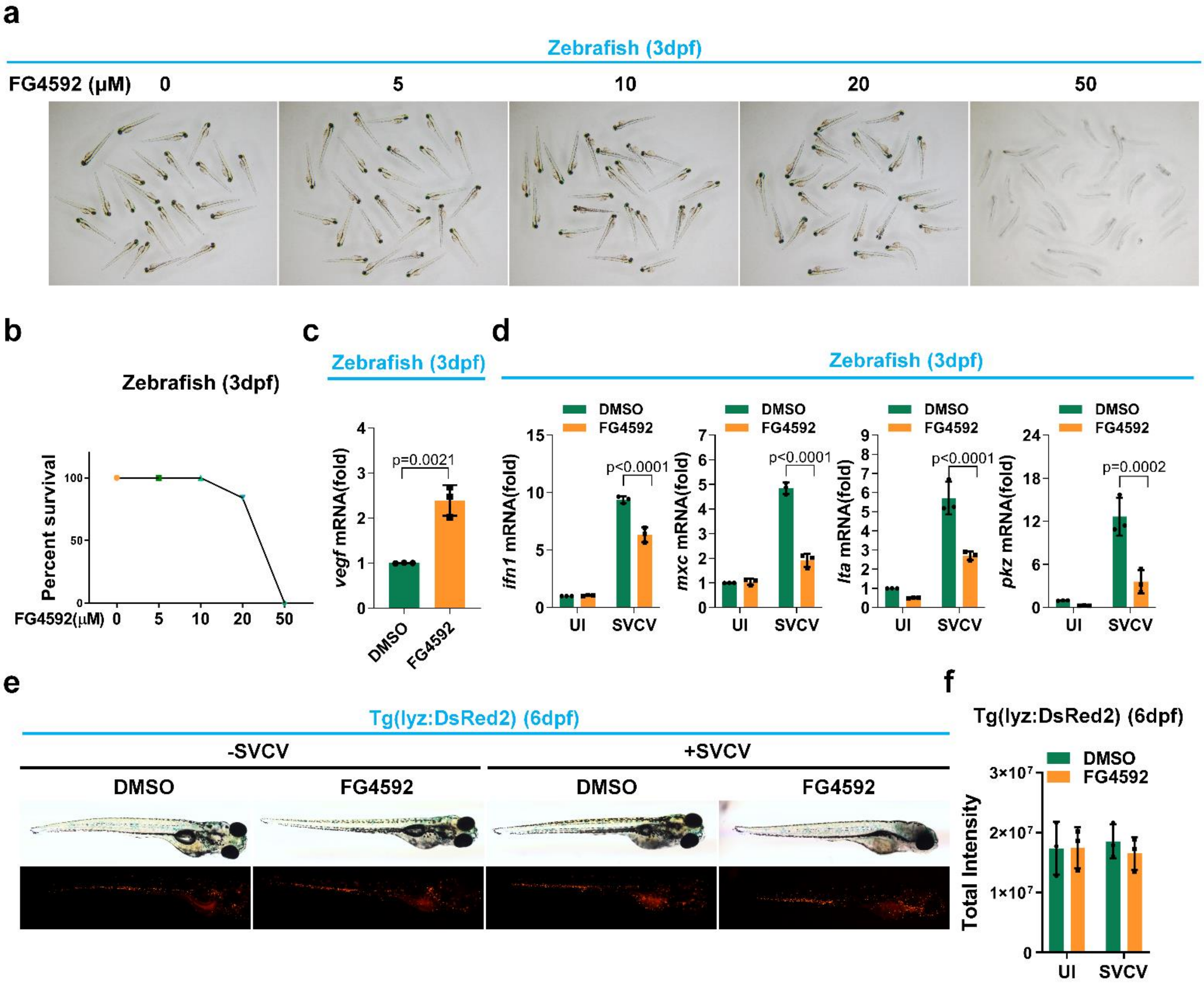

Supplementary Fig. 8. Inhibition of egln1 activity in zebrafish by FG4592 results in increased susceptibility to lethal viral infection. Related to Fig. 4.

(a) Representative images of zebrafish larvae (3 days post fertilization; 3 dpf) treated with FG4592 (from o to 50 μM) for 24 h. (b) Quantitation of percent survival in (a). (c) qPCR analysis of *vegf* mRNA in zebrafish larvae (3 dpf) treated with DMSO (vehicle control) or FG4592 (10 μM) for 12 h. (d) qPCR analysis of *ifn1*, *mxc*, *lta* and *pkz* mRNA in zebrafish larvae (3 dpf) treated with DMSO (vehicle control) or FG4592 (10 μM) for 12 h, followed by uninfected (UI) or infected with SVCV (~6 $\times 10^7$ $TCID_{50}$/ml) for 24 h. (e) Representative images of Tg(lyz: DsRed2) zebrafish larvae (6 dpf) treated with DMSO (vehicle control) or FG4592 (10 μM) for 12 h, followed by infected without (-) or with SVCV (~6 $\times 10^7$ $TCID_{50}$/ml) for 24 h. (f) Quantitation of total intensity in (e). UI, uninfected. Data in (**a, e**) are representative from three independent experiments. Data in (**c**) are presented as mean ± S.D., two-tailed student's *t* test; *n*=3 biological independent experiments. Data in (**d**) are presented as mean ± S.D., two-way ANOVA; *n*=3 biological independent experiments. Source data are provided as a Source Data file.

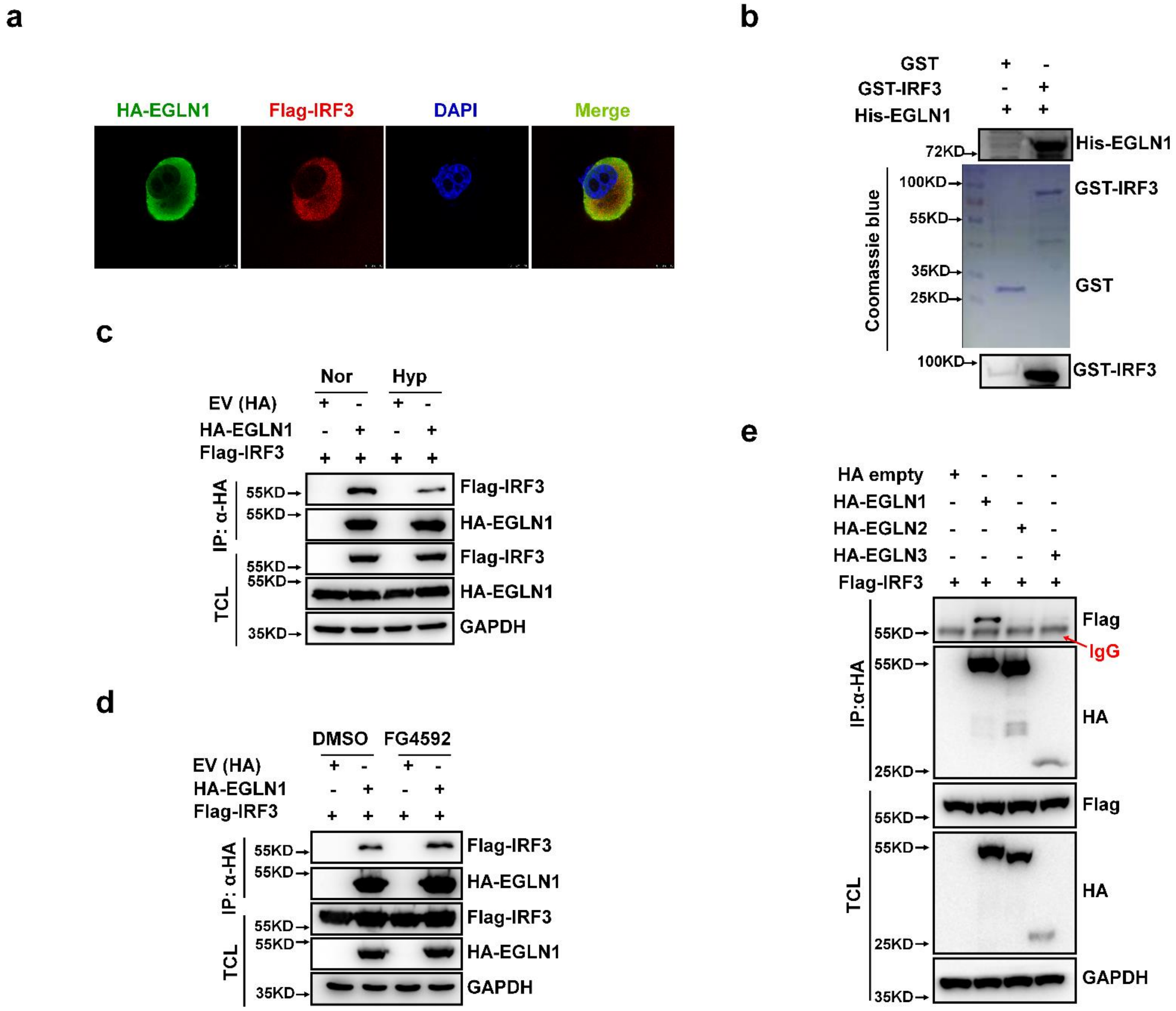

Supplementary Fig. 9. EGLN1 interacts with IRF3. Related to Fig. 5.

(a) Co-localization of EGLN1 and IRF3. Flag-IRF3 and HA-EGLN1 were co-transfected into H1299 cells for 24 h. Confocal microscopy image of Flag-IRF3 was detected by immunofluorescence staining with anti-Flag antibody and HA-tagged EGLN1 was detected by immunofluorescence staining with anti-HA antibody. (b) GST pull-down assay for GST-tagged IRF3 and His-tagged EGLN1. GST-tagged IRF3 and His-tagged EGLN1 were expressed in *Escherichia coli* (BL21), respectively. The association of GST-IRF3 with His-EGLN1 was detected by immunoblotting with anti-EGLN1 and anti-IRF3 antibodies. GST and GST-IRF3 proteins were stained with Coomassie blue. (c) Co-immunoprecipitation of Flag-IRF3 with HA-EGLN1 under normoxia (21% O_2) or hypoxia (1% O_2). HEK293T cells were co-transfected with the indicated plasmids for 18-24 h, followed by treatment under normoxia or hypoxia for 4 h. Anti-HA antibody conjugated agarose beads were used for immunoprecipitation and the interaction was detected by immunoblotting with anti-Flag antibody. (d) Co-immunoprecipitation of Flag-IRF3 with HA-EGLN1 in the presence of DMSO (vehicle control) or FG4592 (20 µM). HEK293T cells were co-transfected with the indicated plasmids for 24 h, followed by treatment with DMSO or FG4592 (20 µM) for 6 h. Anti-HA antibody conjugated agarose beads were used for immunoprecipitation and the interaction was detected with anti-Flag antibody. (e) Co-immunoprecipitation analysis of Flag-IRF3 with HA-EGLN1, HA-EGLN2, and HA-EGLN3. HEK293T cells were co-transfected with the indicated plasmids. Anti-HA antibody conjugated agarose beads were used for

immunoprecipitation and the interaction was analyzed by immunoblotting with anti-Flag antibody. EV, empty vector; IP, immunoprecipitation; TCL, total cell lysates. Data in (**a-e**) are representative from three independent experiments. Source data are provided as a Source Data file.

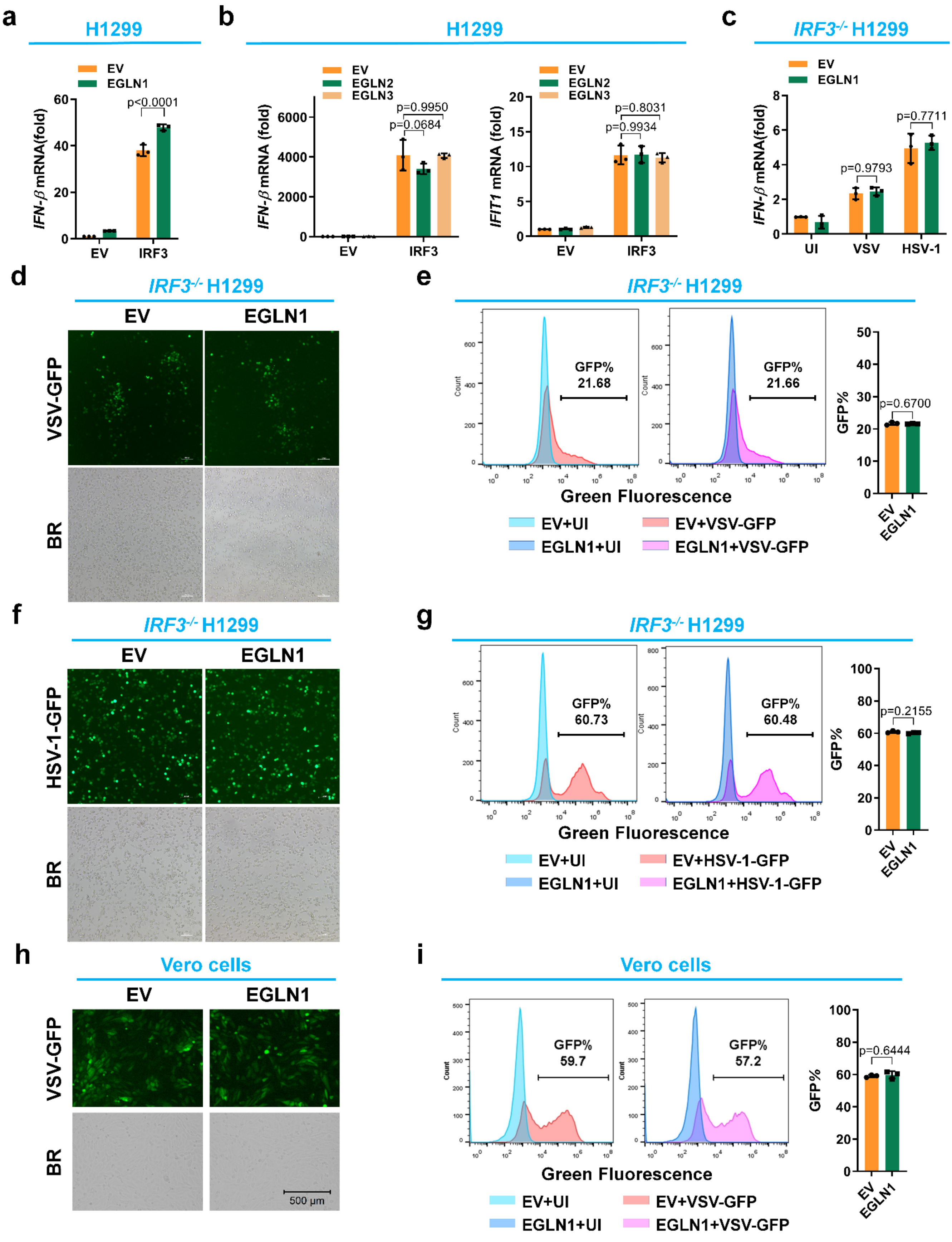

Supplementary Fig. 10. *EGLN1* promotes cellular antiviral immune response dependent on IRF3 and type I IFN. Related to Fig. 5.

(a) qPCR analysis of *IFN-β* mRNA in H1299 cells transfected with the Myc empty vector (EV) or Myc-tagged *IRF3*, together with the HA empty vector (EV) or HA-tagged *EGLN1* (HA-*EGLN1*). (b) qPCR analysis of *IFN-β* and *IFIT1* mRNA in H1299 cells transfected with the Myc empty vector (EV) or Myc-tagged *IRF3*, together with the HA empty vector, or HA-*EGLN2*, or HA-*EGLN3*. (c) qPCR analysis of *IFN-β* mRNA in *IRF3*$^{-/-}$ H1299 cells transfected with the HA empty vector (EV) or HA-*EGLN1*, followed by uninfected (UI) or infected with VSV or HSV-1 for 8 h. (d, e) *IRF3*$^{-/-}$ H1299 cells were transfected with the HA empty vector (EV) or HA-tagged *EGLN1* for 24 h, followed by infection without (UI) or with VSV-GFP virus for 12 h, and viral infectivity was detected by fluorescence microscopy (d) or flow cytometry analysis (e). (f, g) *IRF3*$^{-/-}$ H1299 cells were transfected with the HA empty vector (EV) or HA-*EGLN1* for 24 h, followed by infection without (UI) or with HSV-1-GFP viruses for 12 h, and viral infectivity was detected by fluorescence microscopy (f) or flow cytometry analysis (g). (h, i) Vero cells were transfected with the HA empty vector (EV) or HA-tagged *EGLN1* for 24 h, followed by infection without (UI) or with VSV-GFP viruses for 12 h, and viral infectivity was detected by fluorescence microscopy (h) or flow cytometry analysis (i). EV, empty vector; UI, uninfected. BR, bright field. Data in (**a-c**) are presented as mean ± S.D., two-way ANOVA; *n*=3 biological independent experiments. Data in (**d-i**) are representative from three independent experiments. Data in (**e, g, i**) are presented as mean ± S.D., two-tailed student's *t* test; *n*=3 biological independent experiments. Source data are provided as a Source Data file.

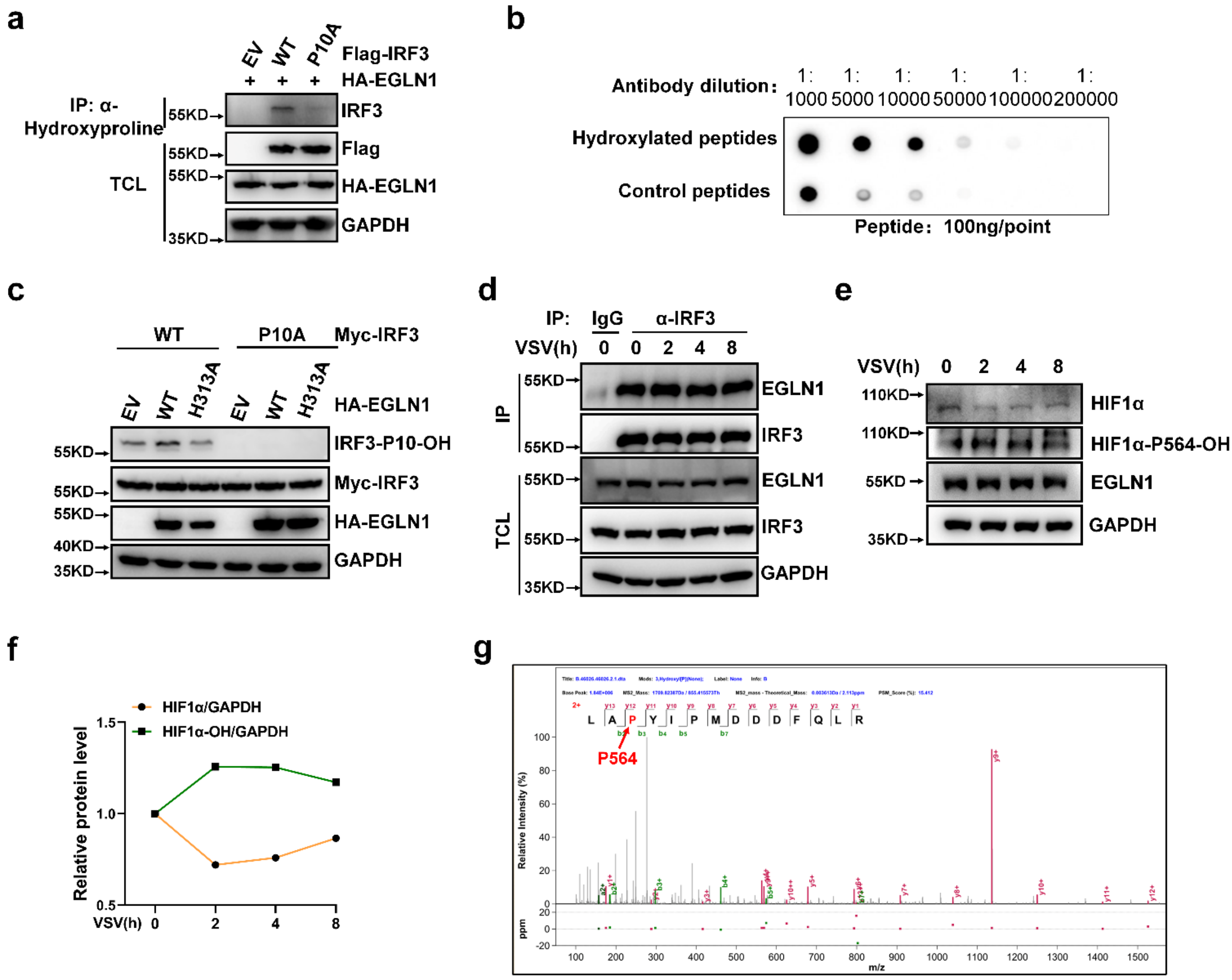

Supplementary Fig. 11. EGLN1 hydroxylates IRF3 at proline 10. Related to Fig. 6.

(a) HEK293T cells were transfected with the Flag empty vector (EV), Flag-IRF3 or Flag-IRF3-P10A, followed by immunoprecipitation with anti-hydroxyproline antibody, and immunoblotting with anti-IRF3 antibody. (b) Dot blot assay for the specificity of anti-IRF3-P10-OH antibody. Equal amounts of hydroxylated peptides or the control peptides were immunoblotted with the indicated dilutions of anti-IRF3-P10-OH antibody. (c) HEK293T cells were transfected with Myc-IRF3-WT or Myc-IRF3-P10A, together with the HA empty vector (EV), HA-EGLN1-WT (WT), HA-EGLN1-H313A (H313A), followed by immunoprecipitation with anti-Myc antibody, and immunoblotting with anti-IRF3-P10-OH antibody. (d) Cell lysates from H1299 cells infected with VSV virus for the indicated time were extracted, followed by immunoprecipitation with anti-IRF3 antibody, and immunoblotting with anti-EGLN1 and anti-IRF3 antibodies. (e) Cell lysates from H1299 cells infected with VSV virus for the indicated time were extracted, followed by immunoblotting with anti-HIF1α and anti-HIF1α-P564-OH antibodies. (f) Quantitation of the intensity of HIF1α and HIF1α-OH protein is shown in (e). (g) Bacterially expressed HIF1α-ODD (400-575aa) (GST tag has been removed using thrombin) was incubated with bacterially expressed GST-EGLN1 for hydroxylation reaction and then identified by mass spectrometry analysis. Hydroxylated HIF1α-P564 is indicated by the red arrow. EV, empty vector; IP, immunoprecipitation; TCL, total cell lysates. Data in (**a-e**) are representative from three independent experiments. Source data are provided as a Source Data file.

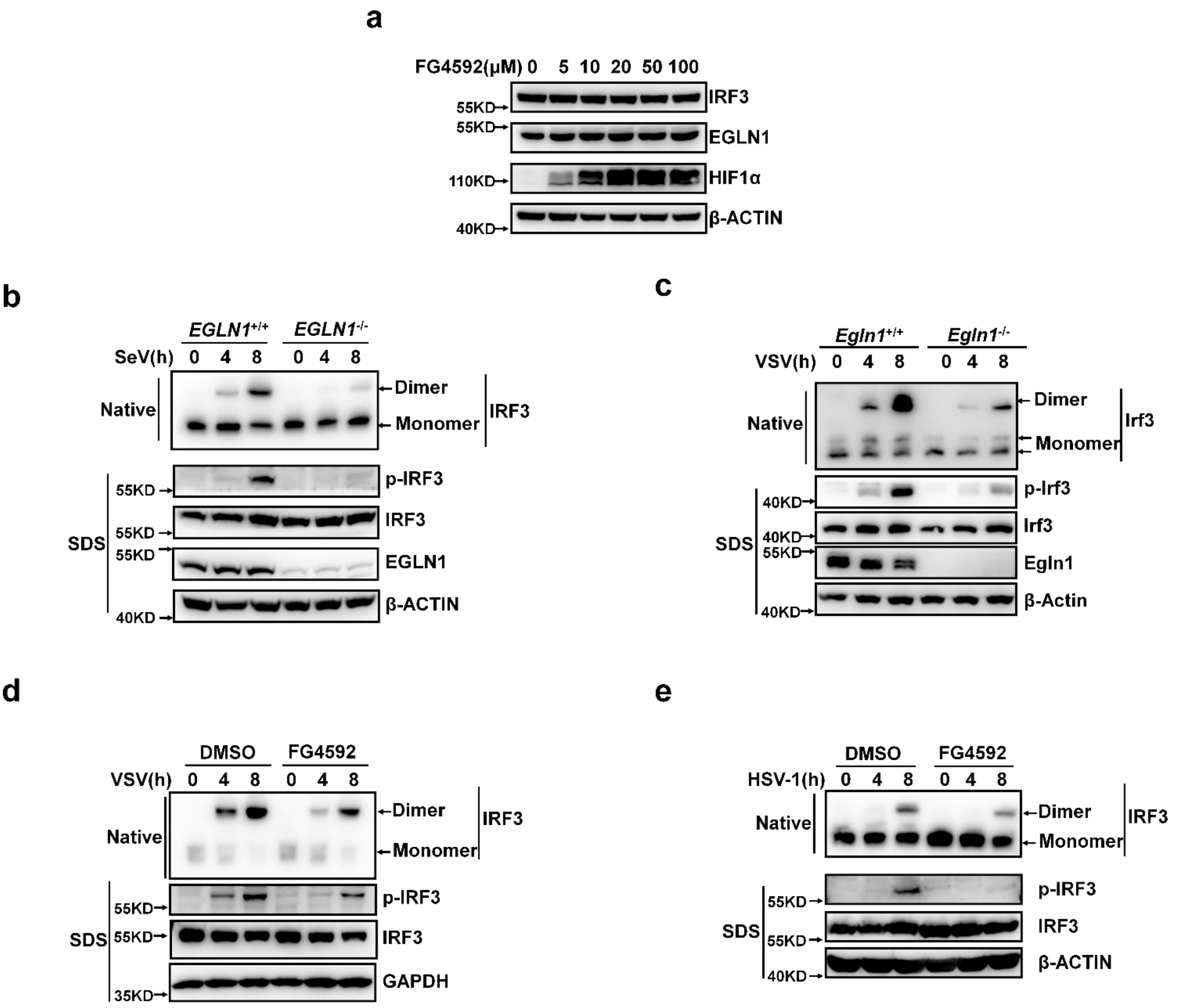

Supplementary Fig. 12. EGLN1 enhances IRF3 phosphorylation and dimerization. Related to Fig. 7.

(a) Immunoblotting of the indicated protein expression in H1299 cells treated with FG4592 at different concentrations (from 0 to 100 μM) for 6 h. (b) *EGLN1*$^{+/+}$ and *EGLN1*$^{-/-}$ H1299 cells were infected with SeV for the indicated times, and the cell lysates were analyzed by immunoblotting for monomeric (Monomer) and dimeric (Dimer) IRF3 (top; native-PAGE); phosphorylated IRF3 (p-IRF3), total IRF3, EGLN1, and β-ACTIN (bottom; SDS-PAGE). (c) *Egln1*$^{+/+}$ and *Egln1*$^{-/-}$ MEF cells were infected with VSV for the indicated times, and the cell lysates were analyzed by immunoblotting for monomeric (Monomer) and dimeric (Dimer) Irf3 (top; native-PAGE); phosphorylated Irf3 (p-Irf3), total Irf3, Egln1, and β-Actin (bottom; SDS-PAGE). (d) H1299 cells were treated with DMSO (vehicle control) or FG4592 (20 μM) for 6 h, followed by infection with VSV for the indicated times, and cell lysates were analyzed by immunoblotting for monomeric (monomer) and dimeric (dimer) IRF3 (top, native-PAGE); phosphorylated IRF3 (p-IRF3), total IRF3, and GAPDH (bottom, SDS-PAGE). (e) H1299 cells were treated with DMSO (vehicle control) or FG4592 (20 μM) for 6 h, followed by infection with HSV-1 for the indicated times, and cell lysates were analyzed by immunoblotting for monomeric (monomer) and dimeric (dimer) IRF3 (top, native-PAGE); phosphorylated IRF3 (p-IRF3), total IRF3, and β-ACTIN (bottom; SDS-PAGE). Data in (**a-e**) are representative from three independent experiments. Source data are provided as a Source Data file.

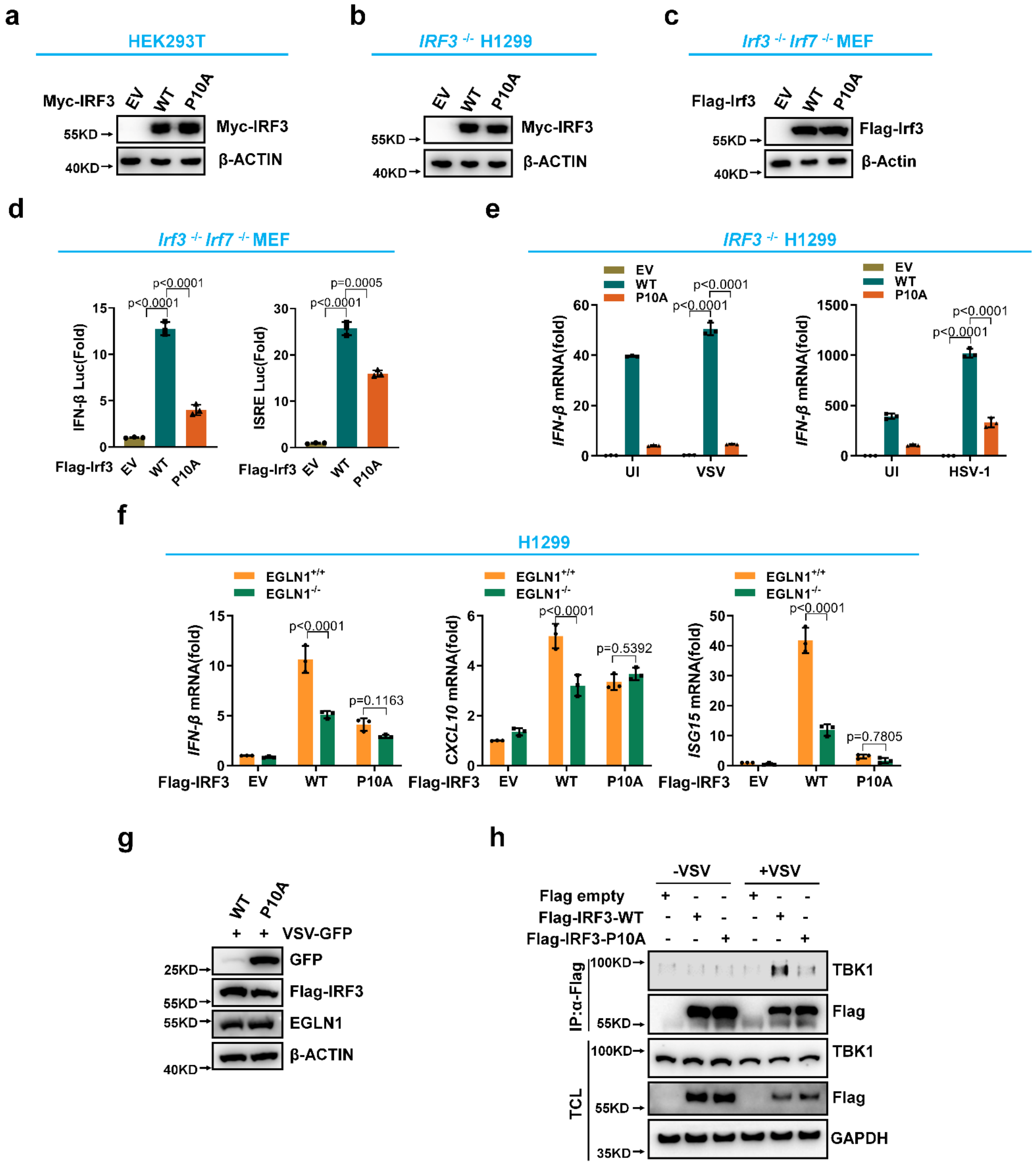

Supplementary Fig. 13. Hydroxylation of IRF3 at proline 10 enhances IRF3 activation in cellular antiviral immune responses. Related to Fig. 8.

(a, b) Validation of indicated protein expression in HEK293T cells (a) and *IRF3*$^{-/-}$ H1299 cells (b), which were transfected with the empty vector (EV) or the plasmid expressing wild-type IRF3 (WT) or its mutant (P10A). (c) Validation of indicated protein expression in *Irf3* $^{-/-}$ *Irf7* $^{-/-}$ MEF cells transfected with the empty vector (EV) or the plasmid expressing wild-type Irf3 (WT) or its mutant (P10A). (d) IFN-β promoter activity and ISRE reporter activity in *Irf3* $^{-/-}$ *Irf7* $^{-/-}$ MEF cells transfected with the empty vector (EV) or the plasmid expressing wildtype Irf3 (WT) or its

mutant (P10A). (e) qPCR analysis of *IFN-β* mRNA in *IRF3* $^{-/-}$ H1299 cells transfected with the empty vector (EV) or the plasmid expressing wild-type IRF3 (WT) or its mutant (P10A), followed by infection without (UI) or with VSV or HSV-1 for 8 h. (f) qPCR analysis of *IFN-β, CXCL10* and *ISG15* mRNA in *EGLN1* $^{+/+}$ and *EGLN1* $^{-/-}$ H1299 cells transfected with the empty vector (EV) or the plasmid expressing Flag-tagged wild-type IRF3 (WT) or its mutant (P10A). (g) *IRF3* $^{-/-}$H1299 cells were transfected with the plasmid expressing Flag-tagged wild-type IRF3 (WT) or its mutant (P10A) for 24 h, followed by infection without (UI) or with VSV-GFP viruses for 12h, and viral infectivity was detected by immunoblotting with anti-GFP antibody. (h) *IRF3* $^{-/-}$ H1299 cells were transfected with empty vector or the plasmid expressing Flag-tagged wild-type IRF3 (WT) or P10A mutant (P10A), followed by infection without or with VSV for 8 h. Anti-Flag antibody conjugated agarose beads were used for immunoprecipitation and the interaction was analyzed by immunoblotting with anti-TBK1 antibody. IP, immunoprecipitation; TCL, total cell lysates. Data in (**a-c, g, h**) are representative from three independent experiments. Data in (**d**) are presented as mean ± S.D., two-tailed student's *t* test; *n*=3 biological independent experiments. Data in (**e, f**) are presented as mean ± S.D., two-way ANOVA; *n*=3 biological independent experiments. Source data are provided as a Source Data file.

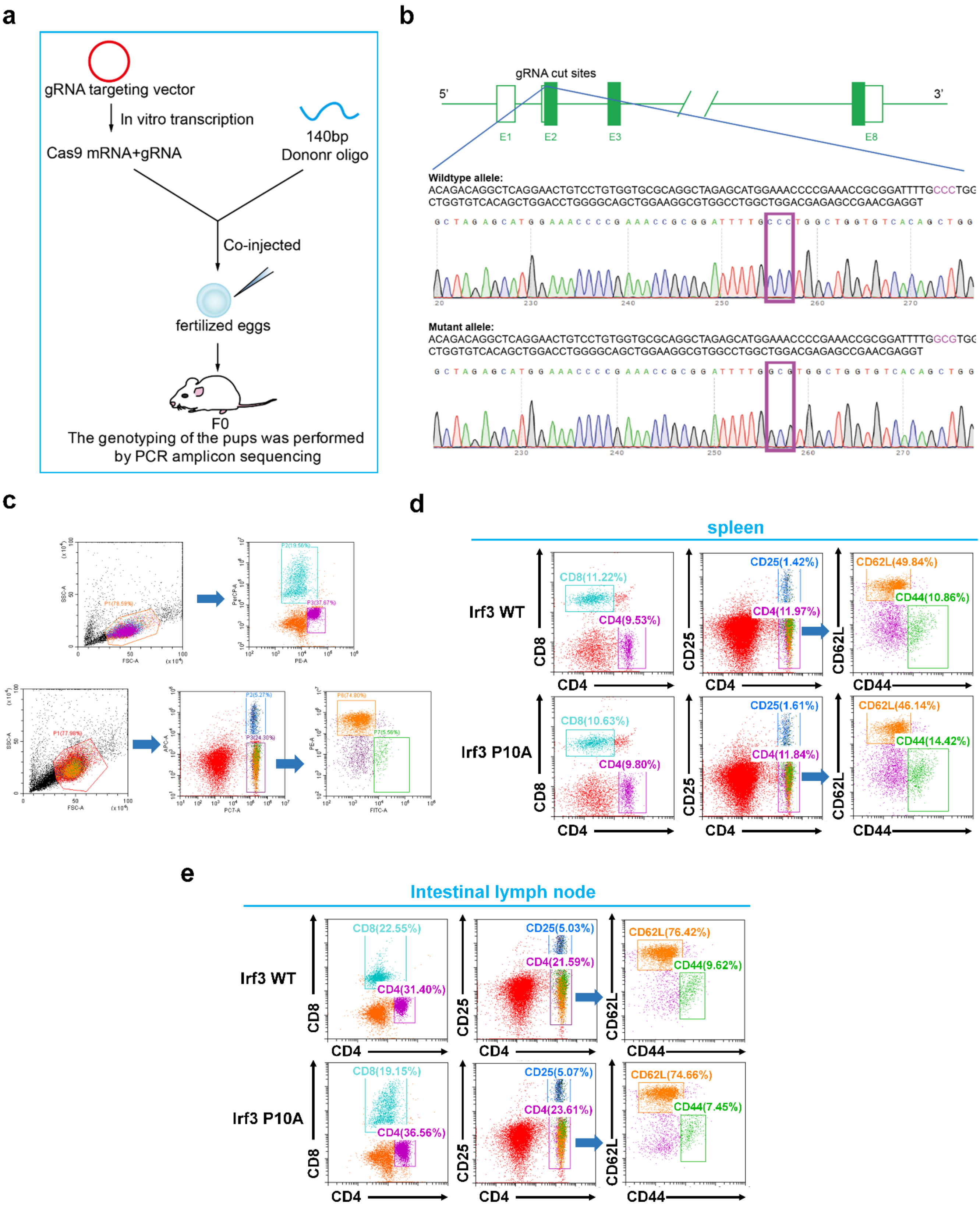

Supplementary Fig. 14. Generation of Irf3_P10A mutant mice. Related to Fig. 9.

(a) Schematic of CRISPR/Cas9-mediated generation of Irf3_P10A mutant mice. (b) Schematic of the resulting nucleotide sequence in Irf3_P10A mutant mice. (c) Gating strategy to determine the percentage of immune cells,

respectively. (d, e) Flow cytometry analysis of cells from spleen and intestinal lymph nodes stained with the indicated antibodies. Data in (**d, e**) are representative from three independent experiments.

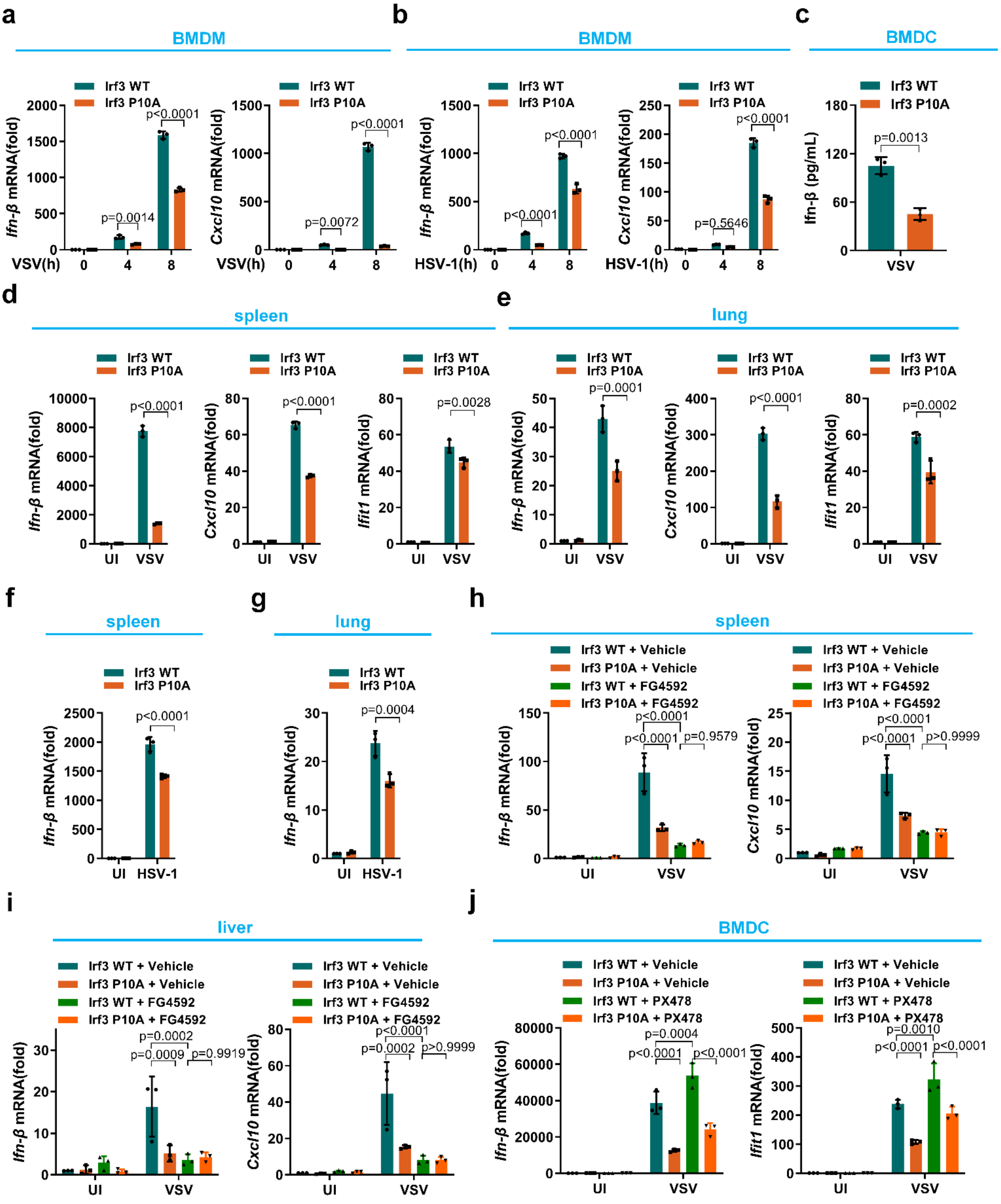

Supplementary Fig. 15. Irf3 prolyl hydroxylation deficiency attenuates antiviral innate immunity in mice. Related to Fig. 9.

(a) qPCR analysis of *Ifn-β* and *Cxcl10* mRNA in WT or *Irf3*_P10A mutant BMDMs (*Irf3*-WT or *Irf3*_P10A) infected with VSV for 0, 4 and 8 h. (b) qPCR analysis of *Ifn-β* and *Cxcl10* mRNA in WT or *Irf3*_P10A mutant BMDMs (*Irf3*-WT or *Irf3*_P10A) infected with HSV-1 for 0, 4 and 8 h. (c) ELISA assay of Ifn-β in the supernatant of WT or

*Irf3*_P10A mutant BMDCs (*Irf3*-WT or *Irf3*_P10A) infected without (UI) or with VSV for 8 h. (d, e) qPCR analysis of *Ifn-β*, *Cxcl10* and *Ifit1* mRNA in spleen (d) or lung (e) of WT or *Irf3*_P10A mutant mice (*Irf3*-WT or *Irf3*_P10A) injected intraperitoneally with PBS (vehicle control) (UI) or VSV (1×10^7 PFU per mouse) for 24 h. (f, g) qPCR analysis of *Ifn-β* mRNA in spleen (f) or lung (g) of WT or *Irf3*_P10A mutant mice (*Irf3*-WT or *Irf3*_P10A) injected intraperitoneally with PBS (vehicle control) (UI) or HSV-1 (1×10^7 PFU per mouse) for 24 hrs. (h, i) qPCR analysis of *Ifn-β,* and *Cxcl10* mRNA in spleen (h) or liver (i) form WT or *Irf3*_P10A mutant mice (*Irf3*-WT or *Irf3*_P10A) treated with DMSO (vehicle control) or FG4592, followed by intraperitoneal injection of PBS(UI) or VSV (1×10^7 PFU per mouse) for 24 h. (j) qPCR analysis of *Ifn-β and Ifit1* mRNA in WT or *Irf3*_P10A mutant BMDCs (*Irf3*-WT or *Irf3*_P10A) treated with DMSO or PX478 (10 μM), followed by infection without (UI) or with VSV (1×10^7 PFU per mouse) for 8 h. UI, uninfected. Data in (**a, b, d-j**) are presented as mean ± S.D., two-way ANOVA; *n*=3 biological independent experiments. Data in (**c**) are presented as mean ± S.D., two-tailed student's *t* test; *n*=3 biological independent samples. Source data are provided as a Source Data file.

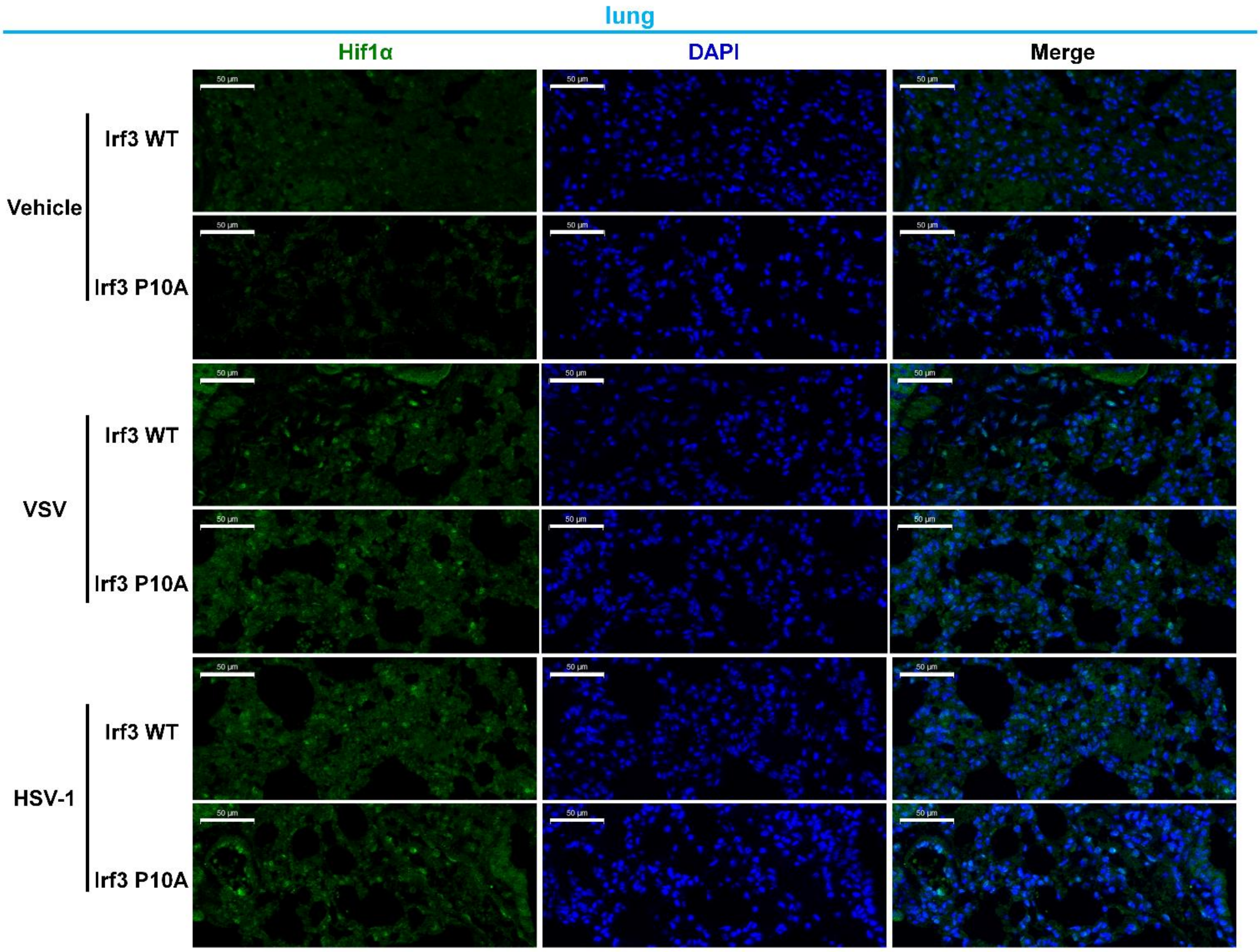

Supplementary Fig. 16. Detection of HIF1α protein in wild-type and Irf3_P10A mutant mice after virus infection. Related to Fig. 9.

Immunofluorescence images of HIFα protein in the lungs of *Irf3*-WT and *Irf3*_P10A mutant mice injected intraperitoneally injected with vehicle control (PBS), VSV (5×10^7 PFU per mouse) or HSV-1 (5×10^7 PFU per mouse) for 24 hours. Data are representative from three independent experiments. Source data are provided as a Source Data file.

.

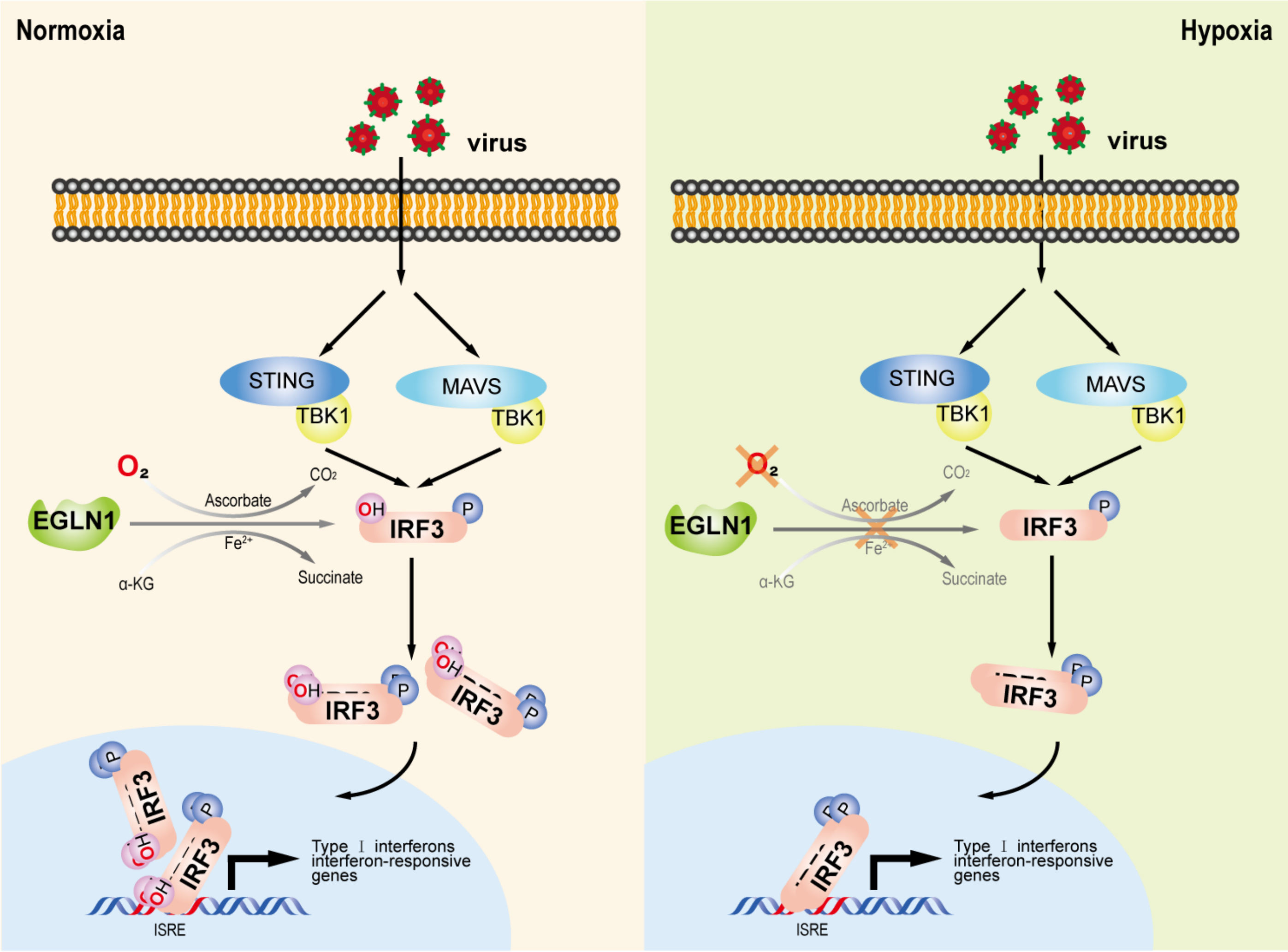

Supplementary Fig. 17. A working model of oxygen in antiviral innate immunity. Under normoxia, upon viral infection, due to sufficient oxygen, the activity of EGLN1 is sustained activated, leading to IRF3 hydroxylation at proline 10. IRF3 prolyl hydroxylation promotes IRF3 phosphorylation, dimerization and nuclear translocation and subsequent activation, resulting in antiviral innate immune responses by transactivating type I interferon gene expression and subsequent interferon-stimulated gene expression. Under hypoxia, oxygen deprivation, the activity of EGLN1 is switched off, leading to the abrogation of IRF3 prolyl hydroxylation. This leads to a reduction in IRF3 phosphorylation, dimerization, nuclear translocation, and subsequent activation.

Supplementary Table 1. Reagents.

Reagents	Source	Identifier
**Chemicals**		
poly I:C	Invivogen	Cat#tlrl-pic-5
DMSO	Sigma-Aldrich	Cat#D2650
DMOG	Sigma-Aldrich	Cat#400091
DAPI	Thermo Fisher	Cat#62248
Transfection Reagent VigoFect	Vigorous Biotech	Cat#T001
Lipofectamine 2000 Reagent	Thermo Fisher	Cat#1668019
Protease Inhibitor Cocktail	Bimake	Cat#B14001
Phosphatase Inhibitor Cocktail	Cell Signaling Technology	Cat#5870S
RNAiso plus	Takara	Cat#9109
SYBR Green qRCR Mix(High ROX)	Monad Biotech	Cat#MQ10301S
Recombinant Human GM-CSF	PeproTech	Cat#315-03
Recombinant Human M-CSF	PeproTech	Cat#315-02
**Antibodies**		
Rabbit monoclonal anti-EGLN1	Cell Signaling Technology	Cat#4835
Rabbit monoclonal anti-IRF3	Cell Signaling Technology	Cat#4302
Rabbit monoclonal anti-Phospho-IRF3 (Ser396)	Cell Signaling Technology	Cat# 29047
Rabbit monoclonal anti-HIF1α	Cell Signaling Technology	Cat#36169
Rabbit monoclonal anti-ARNT	Cell Signaling Technology	Cat#5537
Rabbit monoclonal anti-Histone H3	Cell Signaling Technology	Cat#4499
Rabbit monoclonal anti-TBK1	Cell Signaling Technology	Cat#3504
Rabbit monoclonal anti-HA	Cell Signaling Technology	Cat#3724
Rabbit monoclonal anti-IRF3	Cell Signaling Technology	Cat#11904
Mouse monoclonal anti-HA	Covance	Cat#901515
Mouse monoclonal anti-Myc	Santa Cruz	Cat# sc-40
Mouse monoclonal anti-Flag	Sigma-Aldrich	Cat#F1804
Mouse monoclonal anti-GAPDH	Santa Cruz	Cat#sc-47724
Mouse monoclonal anti-GFP	ABclonal	Cat#AE012
Rabbit polyclonal anti-β-actin	ABclonal	Cat#AC026
Rabbit polyclonal anti-Hydroxyproline	Abcam	Cat#ab37067
Rabbit polyclonal anti-IRF3-P10-OH	ABclonal	N/A

Supplementary Table 2. **The primer sequences**

Primers	Sequence (5' to 3')
Human-*GAPDH*-RT-F	GGAGAAACCTGCCAAGTATG
Human-*GAPDH*-RT-R	CATACCAGGAAATGAGCTTG
Human-*PDK1*-RT-F	ACCAGGACAGCCAATACAAG
Human-*PDK1*-RT-R	CCTCGGTCACTCATCTTCAC
Human-*PGK1*-RT-F	TGGCTTCTGGCATACCTGCT
Human-*PGK1*-RT-R	GCTGCTTTCAGGACCACAGCT
Human-*LDHA*-RT-F	ATCTTGACCTACGTGGCTTGGA
Human-*LDHA*-RT-R	CCATACAGGCACACTGGAATCTC
Human-*GLUT1*-RT-F	CGGGCCAAGAGTGTGCTAAA
Human-*GLUT1*-RT-R	TGACGATACCGGAGCCAATG
Human-*VEGF*-RT-F	CTTGCCTTGCTGCTCTAC
Human-*VEGF*-RT-R	TGGCTTGAAGATGTACTGG
Human-*BNIP3*-RT-F	CTTCCATCTCTGCTGCTCTC
Human-*BNIP3*-RT-R	GTAATCCACTAACGAACCAAGTC
Human-*IFNβ*-RT-F	ACGCCGCATTGACCATCTATG
Human-*IFNβ*-RT-R	CGGAGGTAACCTGTAAGTCTGT
Human-*CXCL10*-RT-F	GCTCTACTGAGGTGCTATGTTC
Human-*CXCL10*-RT-R	GGAGGATGGCAGTGGAAGTC
Human-*IFIT1*-RT-F	CCTCCTTGGGTTCGTCTACA
Human-*IFIT1*-RT-R	GGCTGATATCTGGGTGCCTA
Human-*ISG15*-RT-F	GAGAGGCAGCGAACTCATCTT
Human-*ISG15*-RT-R	CCAGCATCTTCACCGTCAGG
Human-*CCL5*-RT-F	ATCCTCATTGCTACTGCCCTC
Human-*CCL5*-RT-R	GCCACTGGTGTAGAAATACTCC
Mouse-*β-actin*-RT-F	TGGAATCCTGTGGCATCCATGAAAC
Mouse-*β-actin*-RT-R	TAAAACGCAGCTCAGTAACAGTCCG
Mouse-*Ifnβ*-RT-F	TACAACAGATACGCCTGGAT
Mouse-*Ifnβ*-RT-R	AGTCCGCCTCTGATGCTTAA
Mouse-*Cxcl10*-RT-F	CCAAGTGCTGCCGTCATTTT
Mouse-*Cxcl10*-RT-R	GATAGGCTCGCAGGGATGAT
Mouse-*Ifit1*-RT-F	CCAAGTGTTCCAATGCTCCT
Mouse-*Ifit1*-RT-R	GGATGGAATTGCCTGCTAGA
Mouse-*Isg15*-RT-F	CCTCTGAGCTCCTGGTGAG
Mouse-*Isg15*-RT-R	ACTGGTCTTCGTGGACTTGTT
Mouse-*Ccl5*-RT-F	ATTGTCGGCGTCCACAAAG
Mouse-*Ccl5*-RT-R	GTGCATCGTTGTATTTCCGCA
VSV-RT-F	TGGGATGACTGGGCTCCATA
VSV-RT-R	CACCATCAGGAAGCTGCGAA
Zebrafish-*β-actin*-RT-F	TACAATGAGCTCCGTGTTGC
Zebrafish-*β-actin*-RT-R	ACATACAATGGCAGGGGTGTT
Zebrafish-*ifn1*-RT-F	GAGCACATGAACTCGGTGAA

Zebrafish-*ifn1*-RT-R	TGCGTATCTTGCCACACATT
Zebrafish-*pkz*-RT-F	GGAGCACCGTACAGGACATT
Zebrafish-*pkz*-RT-R	CTCGGGCTTTATTTGCTCTG
Zebrafish-*mxc*-RT-F	GAGGCTTCACTTGGCAACTC
Zebrafish-*mxc*-RT-R	TTGTTCCAATAAGGCCAAGC
Zebrafish-*lta*-RT-F	AAGCCAAACGAAGGTCA
Zebrafish-*lta*-RT-R	AACCCATTTCAGCGATTGTC
Zebrafish-*vegf*-RT-F	TGCTCCTGCAAATTCACACAA
Zebrafish-*vegf*-RT-R	ATCTTGGCTTTTCACATCTGCAA
Zebrafish-*mxc*-RT-R	AGAAGCTGATCTGAAAGCCAAAC
Zebrafish-*egln1a*-KO-test-R	ATTCCTGACCAGGTCATCCATG
Zebrafish-*egln1b*- KO-test-F	ACAGTCGAGCACTTCTCAGAG
Zebrafish-*egln1b*- KO-test-R	GTTACAGTGTCGGATCAGATCG
Mouse-*Irf3*-P10A-test-F	CTCCACTCTTGAAATAGGGTGCCAG
Mouse-*Irf3*-P10A-test-R	ACCTGGCTTTCAGGATTCTGTTCC
Mouse-*Egln1*$^{fl/+}$-test-F	CTTCTGGCATTAGTTGACCACAC
Mouse-*Egln1*$^{fl/+}$-test-R	GAATAAGCCCTCTGAAAAGGAACAC
Mouse-Cre-ER-test-F	GAACGCACTGATTTCGACCA
Mouse-Cre-ER-test-R	GCTAACCAGCGTTTTCGTTC

Original Western blots in Supplementary Figures

Fig. S1a

(i) The samples were separated on one gel, and the membranes were cut and incubated with the indicated antibodies.
(ii) The samples were separated on one gel, and the membranes were cut and incubated with the indicated antibody.

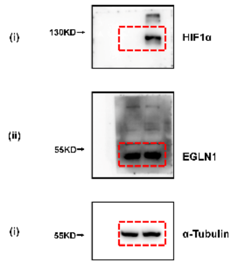

Fig. S1c

(i) The samples were separated on one gel, and the membranes were cut and incubated with the indicated antibodies.
(ii) The samples were separated on one gel, and the membranes were cut and incubated with the indicated antibody.

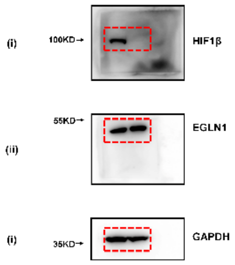

Fig. S1f

(i) The samples were separated on one gel, and the membranes were cut and incubated with the indicated antibodies.

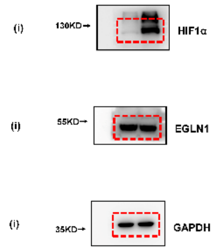

Fig. S2a

(i) The samples were separated on one gel, and the membranes were cut and incubated with the indicated antibodies.
(ii) The samples were separated on one gel, and the membranes were cut and incubated with the indicated antibody.

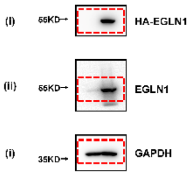

Fig. S2c

(i) The samples were separated on one gel, and the membranes were cut and incubated with the indicated antibodies.

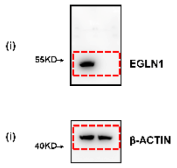

Fig. S2e

(i) The samples were separated on one gel, and the membranes were cut and incubated with the indicated antibodies.

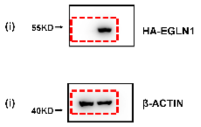

Fig. S2h

(i) The samples were separated on one gel, and the membranes were cut and incubated with the indicated antibodies.

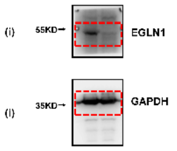

Fig. S2j

(i) The samples were separated on one gel, and the membranes were cut and incubated with the indicated antibodies.

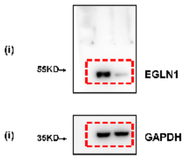

Fig. S2k

(i) The samples were separated on one gel, and the membranes were cut and incubated with the indicated antibodies.

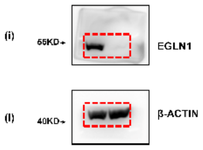

Fig. S2l

(i) The samples were separated on one gel, and the membranes were cut and incubated with the indicated antibodies.

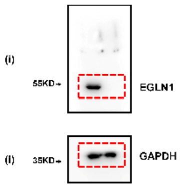

Fig. S3a

(i) The samples were separated on one gel, and the membranes were cut and incubated with the indicated antibodies.

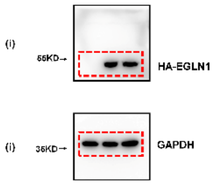

Fig. S3b

(i) The samples were separated on one gel, and the membranes were cut and incubated with the indicated antibodies.

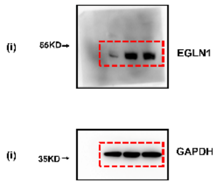

Fig. S3e

(i) The samples were separated on one gel, and the membranes were cut and incubated with the indicated antibody.
(ii) The samples were separated on one gel, and the membranes were cut and incubated with the indicated antibody.

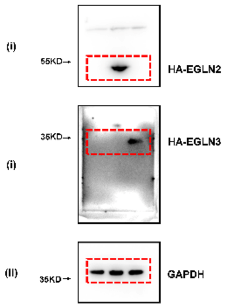

Fig. S5a

(i) The samples were separated on one gel, and the membranes were cut and incubated with the indicated antibody.
(ii) The samples were separated on one gel, and the membranes were cut and incubated with the indicated antibody.

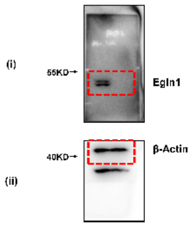

Fig. S6c

(i) The samples were separated on one gel, and the membranes were cut and incubated with the indicated antibodies.

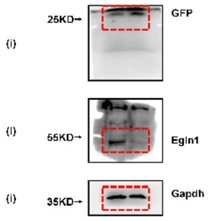

Original Western blots in Supplementary Figures

Fig. S9b

(i) The samples were separated on one gel, and the membranes were cut and incubated with the indicated antibody.
(ii) The samples were separated on one gel, and the gel was stained with Coomassie blue.
(iii) The samples were separated on one gel, and the membranes were cut and incubated with the indicated antibody.

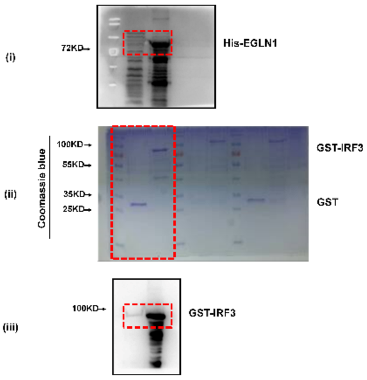

Fig. S9c

(i) The samples were separated on one gel, and the membranes were cut and incubated with the indicated antibody.
(ii) The samples were separated on one gel, and the membranes were cut and incubated with the indicated antibody.
(iii) The samples were separated on one gel, and the membranes were cut and incubated with the indicated antibodies.
(iv) The samples were separated on one gel, and the membranes were cut and incubated with the indicated antibody.

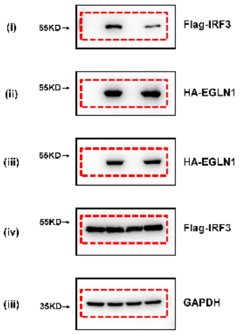

Fig. S9d

(i) The samples were separated on one gel, and the membranes were cut and incubated with the indicated antibody.
(ii) The samples were separated on one gel, and the membranes were cut and incubated with the indicated antibody.
(iii) The samples were separated on one gel, and the membranes were cut and incubated with the indicated antibody.
(iv) The samples were separated on one gel, and the membranes were cut and incubated with the indicated antibodies.

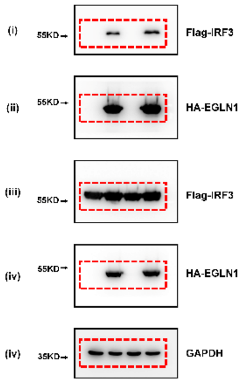

Fig. S9e

(i) The samples were separated on one gel, and the membranes were cut and incubated with the indicated antibody.
(ii) The samples were separated on one gel, and the membranes were cut and incubated with the indicated antibody.
(iii) The samples were separated on one gel, and the membranes were cut and incubated with the indicated antibodies.
(iv) The samples were separated on one gel, and the membranes were cut and incubated with the indicated antibody.

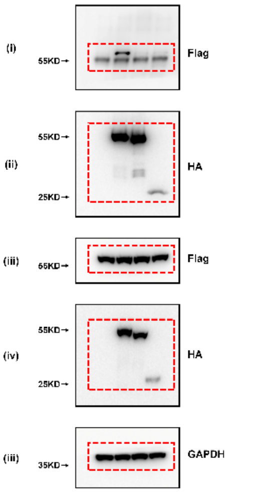

Fig. S11a

(i) The samples were separated on one gel, and the membranes were cut and incubated with the indicated antibody.
(ii) The samples were separated on one gel, and the membranes were cut and incubated with the indicated antibodies.
(iii) The samples were separated on one gel, and the membranes were cut and incubated with the indicated antibody.

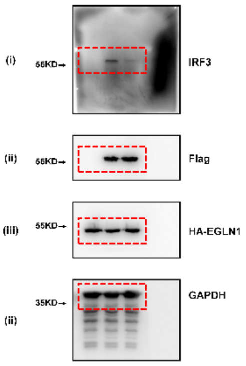

Fig. S11d

(i) The samples were separated on one gel, and the membranes were cut and incubated with the indicated antibody.
(ii) The samples were separated on one gel, and the membranes were cut and incubated with the indicated antibody.
(iii) The samples were separated on one gel, and the membranes were cut and incubated with the indicated antibody.
(iv) The samples were separated on one gel, and the membranes were cut and incubated with the indicated antibodies.

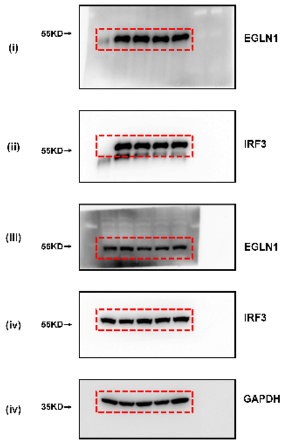

Fig. S11c

(i) The samples were separated on one gel, and the membranes were cut and incubated with the indicated antibody.
(ii) The samples were separated on one gel, and the membranes were cut and incubated with the indicated antibodies.
(iii) The samples were separated on one gel, and the membranes were cut and incubated with the indicated antibody.

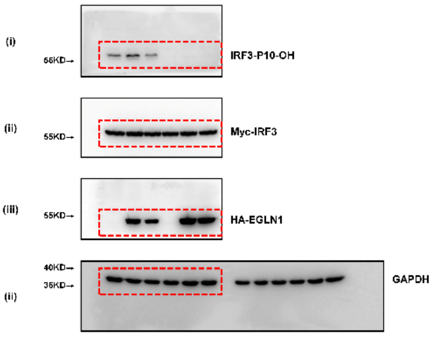

Fig. S11e

(i) The samples were separated on one gel, and the membranes were cut and incubated with the indicated antibody.
(ii) The samples were separated on one gel, and the membranes were cut and incubated with the indicated antibodies.

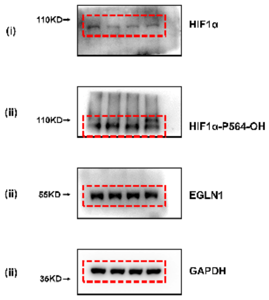

Original Western blots in Supplementary Figures

Fig. S12a

(i) The samples were separated on one gel, and the membranes were cut and incubated with the indicated antibodies.
(ii) The samples were separated on one gel, and the membranes were cut and incubated with the indicated antibody.

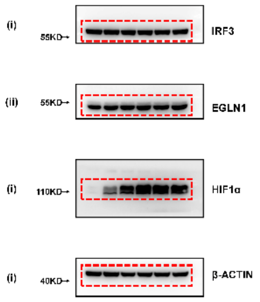

Fig. S12b

(i) The samples were separated on one gel, and the membranes were cut and incubated with the indicated antibody.
(ii) The samples were separated on one gel, and the membranes were cut and incubated with the indicated antibody.
(iii) The samples were separated on one gel, and the membranes were cut and incubated with the indicated antibody.
(iv) The samples were separated on one gel, and the membranes were cut and incubated with the indicated antibodies.

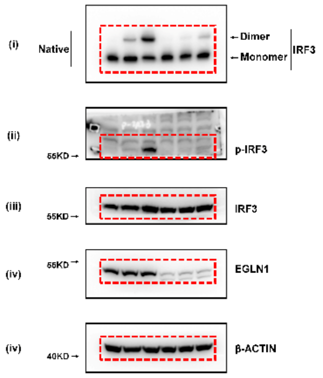

Fig. S12c

(i) The samples were separated on one gel, and the membranes were cut and incubated with the indicated antibody.
(ii) The samples were separated on one gel, and the membranes were cut and incubated with the indicated antibody.
(iii) The samples were separated on one gel, and the membranes were cut and incubated with the indicated antibody.
(iv) The samples were separated on one gel, and the membranes were cut and incubated with the indicated antibody.
(v)The samples were separated on one gel, and the membranes were cut and incubated with the indicated antibody.

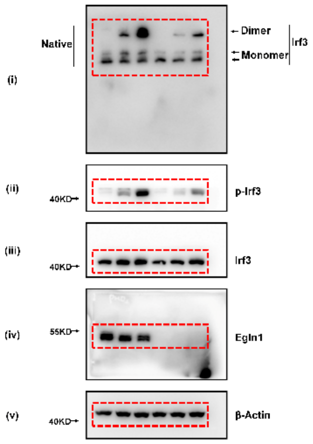

Fig. S12d

(i) The samples were separated on one gel, and the membranes were cut and incubated with the indicated antibody.
(ii) The samples were separated on one gel, and the membranes were cut and incubated with the indicated antibody.
(iii) The samples were separated on one gel, and the membranes were cut and incubated with the indicated antibody.

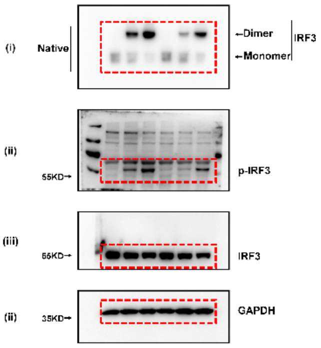

Fig. S12e

(i) The samples were separated on one gel, and the membranes were cut and incubated with the indicated antibody.
(ii) The samples were separated on one gel, and the membranes were cut and incubated with the indicated antibody.
(iii) The samples were separated on one gel. and the membranes were cut and incubated with the indicated antibodies.

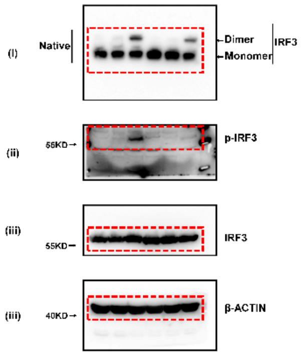

Fig. S13a

(i) The samples were separated on one gel, and the membranes were cut and incubated with the indicated antibodies.

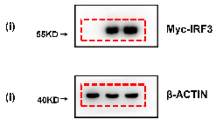

Fig. S13b

(i) The samples were separated on one gel, and the membranes were cut and incubated with the indicated antibodies.

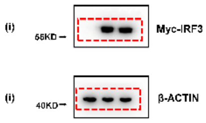

Fig. S13c

(i) The samples were separated on one gel, and the membranes were cut and incubated with the indicated antibodies.

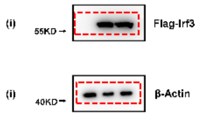

Fig. S13g

(i) The samples were separated on one gel, and the membranes were cut and incubated with the indicated antibodies.
(ii) The samples were separated on one gel, and the membranes were cut and incubated with the indicated antibody.
(iii) The samples were separated on one gel, and the membranes were cut and incubated with the indicated antibody.

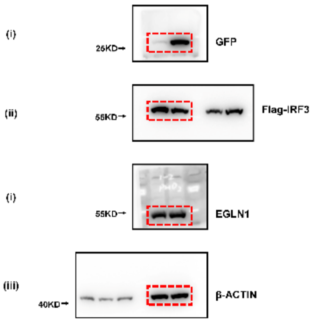

Fig. S13h

(i) The samples were separated on one gel, and the membranes were cut and incubated with the indicated antibodies.
(ii) The samples were separated on one gel, and the membranes were cut and incubated with the indicated antibody.
(iii) The samples were separated on one gel, and the membranes were cut and incubated with the indicated antibodies.

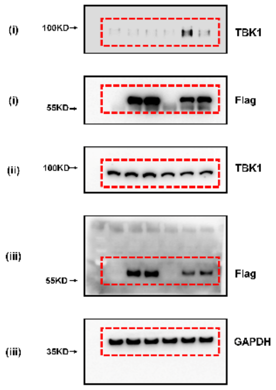

Supplement: Supplementary file 1 — Supplementary Information [file 41467_2024_47814_MOESM1_ESM.pdf]
