# Supplementary material for: Oxygen enhances antiviral innate immunity through maintenance of EGLN1-catalyzed proline hydroxylation of IRF3
Source: Nat Commun. 2024 Apr 26;15:3533. doi: 10.1038/s41467-024-47814-3 (PMC11053110; doi:10.1038/s41467-024-47814-3)
Supplement: Supplementary file 3 — Reporting Summary [file 41467_2024_47814_MOESM3_ESM.pdf]

nature portfolio

Corresponding author(s): Wuhan Xiao

Last updated by author(s): Mar 13, 2024

Reporting Summary

Nature Portfolio wishes to improve the reproducibility of the work that we publish. This form provides structure for consistency and transparency in reporting. For further information on Nature Portfolio policies, see our Editorial Policies and the Editorial Policy Checklist.

Statistics

For all statistical analyses, confirm that the following items are present in the figure legend, table legend, main text, or Methods section.

n/a	Confirmed	
☐	☒	The exact sample size (*n*) for each experimental group/condition, given as a discrete number and unit of measurement
☐	☒	A statement on whether measurements were taken from distinct samples or whether the same sample was measured repeatedly
☐	☒	The statistical test(s) used AND whether they are one- or two-sided<br>*Only common tests should be described solely by name; describe more complex techniques in the Methods section.*
☒	☐	A description of all covariates tested
☐	☒	A description of any assumptions or corrections, such as tests of normality and adjustment for multiple comparisons
☐	☒	A full description of the statistical parameters including central tendency (e.g. means) or other basic estimates (e.g. regression coefficient) AND variation (e.g. standard deviation) or associated estimates of uncertainty (e.g. confidence intervals)
☐	☒	For null hypothesis testing, the test statistic (e.g. *F*, *t*, *r*) with confidence intervals, effect sizes, degrees of freedom and *P* value noted<br>*Give P values as exact values whenever suitable.*
☒	☐	For Bayesian analysis, information on the choice of priors and Markov chain Monte Carlo settings
☒	☐	For hierarchical and complex designs, identification of the appropriate level for tests and full reporting of outcomes
☒	☐	Estimates of effect sizes (e.g. Cohen's *d*, Pearson's *r*), indicating how they were calculated

Our web collection on statistics for biologists contains articles on many of the points above.

Software and code

Policy information about availability of computer code

Data collection	qRT-PCR data were acquired on CFX96TM Real-Time System (BIO-RAD) and collected using Bio-Rad CFX Manager 3.1. Renilla Luciferase activities were collected by SIRIUS Luminometer and Microsoft Excel 2021. Western Blot results were visualized by a Fuji Film LAS4000 mini-luminescent image analyzer using the software Image Reader LAS-4000 2.0. Flow cytometry data were collected using Beckman CytoFLEXS. Immunofluorescent confocal microscopy data were collected using a Leica laser scanning confocal microscope. RNA sequencing was performed on Illumina platforms using the PE150 strategy. Mass spectrometry data were collected on a Q Exactive HF-X mass spectrometer (Thermo Fisher Scientific).
Data analysis	Quantification of Western blot was performed on ImageJ software (version 1.51j8). Immunofluorescence images were analyzed using Leica Application Suite X (LAS X) software (version 3.4.2.18368). Flow cytometry data were generated using CytExpert (version 2.4.0.28) software and analyzed using FlowJo software (version 10.0.7r2). MS/MS spectra analyses were performed using the MaxQuant software and the pFind software (version 3.1). For RNA sequencing, Fastp (version 0.19.7) was used to perform basic statistics on the quality of the raw reads. The RSEM package was used to calculate gene expression levels for each sample, expressed as fragments per kilobase of transcript per million fragments mapped (FPKM). The heat map of expression changes of the indicated genes between different samples was generated using the Multi Experiment Viewer (MeV) software. Gene Ontology (GO) and Kyoto Encyclopedia of Genes and Genomes (KEGG) enrichment analyses for the DEGs were performed using Cluster Profiler version 3.8. Statistical analyses were performed using Micosoft Excel 2021 and GraphPad Prism 8.0 (version8.3.0).

For manuscripts utilizing custom algorithms or software that are central to the research but not yet described in published literature, software must be made available to editors and reviewers. We strongly encourage code deposition in a community repository (e.g. GitHub). See the Nature Portfolio guidelines for submitting code & software for further information.

Data

Policy information about availability of data

All manuscripts must include a data availability statement. This statement should provide the following information, where applicable:

- Accession codes, unique identifiers, or web links for publicly available datasets
- A description of any restrictions on data availability
- For clinical datasets or third party data, please ensure that the statement adheres to our policy

The authors declare that the data supporting the findings of this study are available within the article and its supplementary Information file. Raw mass spectrometry data have been deposited to the ProteomeXchange Consortium (http://proteomecentral.proteomexchange.org) via iProX partner repository with the dataset identifier PXD050566 and PXD048667. The original RNA-seq data were uploaded to the GEO Datasets (GEO accession GSE253181). Source data are provided with this paper.

Research involving human participants, their data, or biological material

Policy information about studies with human participants or human data. See also policy information about sex, gender (identity/presentation), and sexual orientation and race, ethnicity and racism.

Reporting on sex and gender	No human research participants involved in this study.
Reporting on race, ethnicity, or other socially relevant groupings	No human research participants involved in this study.
Population characteristics	No human research participants involved in this study.
Recruitment	No human research participants involved in this study.
Ethics oversight	No human research participants involved in this study.

Note that full information on the approval of the study protocol must also be provided in the manuscript.

Field-specific reporting

Please select the one below that is the best fit for your research. If you are not sure, read the appropriate sections before making your selection.

- [x] Life sciences
- [ ] Behavioural & social sciences
- [ ] Ecological, evolutionary & environmental sciences

For a reference copy of the document with all sections, see nature.com/documents/nr-reporting-summary-flat.pdf

Life sciences study design

All studies must disclose on these points even when the disclosure is negative.

Sample size	No statistical method was used to predetermine sample size. The sample size was determined based on standards for experimental cell biology and animal studies, attempting to have a minimum of n = 3 biological replicates with sufficient reproducibility, and based on previous publications and our experience (PMID: 34171297, PMID: 35675783). The exact n numbers used in each experiment are indicated in the figure legends.
Data exclusions	No data were excluded when experiments were conducted successfully (i.e. experimental controls worked).
Replication	Experimental findings are reproducible, confirmed by repeating the experiments independently with proper controls. The information of replication is clearly indicated in the figure legends.
Randomization	No statistical methods were used for randomization. Animals used in this studies were randomly assigned to infections with VSV and HSV-1 virus. For in vitro experiments, mouse BMDCs and BMDMs were isolated from random wild-type or KO mice, then the isolated primary cells were randomly divided into two groups and treated without (control) or with the indicated stimulations.
Blinding	Blinding was not performed because it is not a standard practice for experiments conducted in this study, and all data were generated objectively with replication that showed similar observations.

Reporting for specific materials, systems and methods

We require information from authors about some types of materials, experimental systems and methods used in many studies. Here, indicate whether each material, system or method listed is relevant to your study. If you are not sure if a list item applies to your research, read the appropriate section before selecting a response.

Materials & experimental systems

n/a	Involved in the study
	☒ Antibodies
	☒ Eukaryotic cell lines
☒	☐ Palaeontology and archaeology
	☒ Animals and other organisms
☒	☐ Clinical data
☒	☐ Dual use research of concern
☒	☐ Plants

Methods

n/a	Involved in the study
☒	☐ ChIP-seq
	☒ Flow cytometry
☒	☐ MRI-based neuroimaging

Antibodies

Antibodies used

Primary antibodies:
Rabbit monoclonal anti-EGLN1 (Cell Signaling Technology, Cat#4835, 1:1000 for WB)
Rabbit monoclonal anti-IRF3 (Cell Signaling Technology, Cat#4302, 1:1000 for WB)
Rabbit monoclonal anti-Phospho-IRF3 (Ser396) (Cell Signaling Technology, Cat# 29047, 1:1000 for WB)
Rabbit monoclonal anti-HIF1α (Cell Signaling Technology, Cat#36169, 1:1000 for WB)
Rabbit monoclonal anti-ARNT (Cell Signaling Technology, Cat#5537, 1:1000 for WB)
Rabbit monoclonal anti-Histone H3 (Cell Signaling Technology, Cat#4499, 1:1000 for WB)
Rabbit monoclonal anti-TBK1 (Cell Signaling Technology, Cat#3504, 1:500 for WB)
Rabbit monoclonal anti-HA (Cell Signaling Technology, Cat#3724, 1:1000 for IF)
Rabbit monoclonal anti-IRF3 (Cell Signaling Technology, Cat#11904, 1:500 for IF)
Mouse monoclonal anti-HA (Covance, Cat#901515, 1:5000 for WB)
Mouse monoclonal anti-Myc (Santa Cruz, Cat# sc-40, 1:2000 for WB)
Mouse monoclonal anti-Flag (Sigma-Aldrich, Cat#F18044, 1:2000 for WB, 1:1000 for IF)
Mouse monoclonal anti-GAPDH (Santa Cruz, Cat#sc-47724, 1:1000 for WB)
Mouse monoclonal anti-GFP (ABclonal, Cat#AE012, 1:1000 for WB)
Rabbit polyclonal anti-β-actin (ABclonal, Cat#AC026, 1:1000 for WB)
Rabbit polyclonal anti-Hydroxyproline (Abcam, Cat#ab37067, 1:500 for WB)
Rabbit polyclonal anti-IRF3-P10-OH (ABclonal, N/A, 1:500 for WB)
Western Blotting Secondary antibodies:
HRP Goat Anti-Mouse IgG (H+L) (ABclonal, Cat#AS003, 1:10000 for WB)
HRP Goat Anti-Rabbit IgG (H+L) (ABclonal, Cat#AS014, 1:10000 for WB)
Immunofluorescent staining secondary antibodies:
Goat anti-Mouse IgG (H+L) Cross-Adsorbed Secondary Antibody, Alexa Fluor 594 (Thermo, Cat Cat#A-11005, 1:2000 for IF)
Goat anti-Rabbit IgG (H+L) Cross-Adsorbed Secondary Antibody, Alexa Fluor 488(Thermo, Cat Cat#A-11008, 1:2000 for IF)

Validation

All the antibodies were from commercial sources and verified by the manufactures. Validation statements and experiments can be obtained from the following websites:
Rabbit monoclonal anti-EGLN1: https://www.cellsignal.cn/products/primary-antibodies/phd-2-egln1-d31e11-rabbit-mab/4835
Rabbit monoclonal anti-IRF3: https://www.cellsignal.cn/products/primary-antibodies/irf-3-d83b9-rabbit-mab/4302
Rabbit monoclonal anti-Phospho-IRF3 (Ser396): https://www.cellsignal.cn/products/primary-antibodies/phospho-irf-3-ser396-d6o1m-rabbit-mab/29047
Rabbit monoclonal anti-HIF1α: https://www.cellsignal.cn/products/primary-antibodies/hif-1a-d1s7w-xp-rabbit-mab/36169
Rabbit monoclonal anti-ARNT: https://www.cellsignal.cn/products/primary-antibodies/hif-1b-arnt-d28f3-xp-174-rabbit-mab/5537
Rabbit monoclonal anti-Histone H3: https://www.cellsignal.cn/products/primary-antibodies/histone-h3-d1h2-xp-rabbit-mab/4499
Rabbit monoclonal anti-TBK1: https://www.cellsignal.cn/products/primary-antibodies/tbk1-nak-d1b4-rabbit-mab/3504
Rabbit monoclonal anti-HA: https://www.cellsignal.cn/products/primary-antibodies/ha-tag-c29f4-rabbit-mab/3724
Rabbit monoclonal anti-IRF3: https://www.cellsignal.cn/products/primary-antibodies/irf-3-d6i4c-xp-174-rabbit-mab/11904
Mouse monoclonal anti-HA: https://www.biolegend.com/en-us/products/anti-ha-11-epitope-tag-antibody-11071
Mouse monoclonal anti-Myc: https://www.scbt.com/zh/p/c-myc-antibody-9e10
Mouse monoclonal anti-Flag: https://www.sigmaaldrich.cn/CN/zh/product/sigma/f1804
Mouse monoclonal anti-GAPDH: https://www.scbt.com/zh/p/gapdh-antibody-0411
Mouse monoclonal anti-GFP: https://abclonal.com.cn/catalog/AE012
Rabbit polyclonal anti-β-actin: https://abclonal.com.cn/catalog/AC026
Rabbit polyclonal anti-Hydroxyproline: validated in Abcam Cat#ab37067 datasheets and in this study by Western blotting analysis (Fig. S11a)
Rabbit polyclonal anti-IRF3-P10-OH: An IRF3 P10 site-specific hydroxylation antibody (anti-IRF3-P10-OH) was generated by using a human IRF3 hydroxylated peptide [C-KPRIL(P-OH)WLVSQLD] as an antigen. After purification of antibodies with excess unmodified peptide (C-KPRILPWLVSQLD), antibodies recognizing site-specific hydroxylation were enriched by biotin-labelled IRF3-hydroxylated peptides. The specificity of the anti-IRF3-P10-OH antibody was verified by dot blot (Fig. S11b) and Western blotting analyses (Fig. 6c-h, 6l, S11c, 9e, 9f).

Eukaryotic cell lines

Policy information about cell lines and Sex and Gender in Research

Cell line source(s)	HEK293T, H1299, THP-1, Vero, and Epithelioma papulosum cyprini (EPC) cell lines were originally obtained from ATCC (American Type Culture Collection). The 780-O cell line was a gift from Dr. William Kaelin. RCC4 cell line was a gift from Dr. Peter Ratcliffe. Irf3-/-Irf7-/- and p65 -/- MEF cells were provided by Dr. Bo Zhong (Wuhan University, China).
Authentication	None of the cell lines used were authenticated.
Mycoplasma contamination	We confirmed that all cell lines were tested negative for Mycoplasma contamination.
Commonly misidentified lines (See ICLAC register)	No commonly misidentified lines were used in this study.

Animals and other research organisms

Policy information about studies involving animals; ARRIVE guidelines recommended for reporting animal research, and Sex and Gender in Research

Laboratory animals	C57BL/6J-Egln1em1cyagen mice were obtained from Cyagen Biosciences, which were generated by CRISPR/Cas9-mediated genome editing. Two loxP sites flank the exons 2 and 3 of the mouse Egln1 gene. Cre-ER mice (B6.129-Gt(ROSA)26Sortm1(cre/ERT2)Tyj) (hereafter referred to as Cre-ER) originally obtained from The Jackson Laboratory were kindly provided by Dr. Bo Zhong (Wuhan University). Egln1fl/+ mice were crossed with Cre-ER mice to generate Cre-ER Egln1fl/+ mice. After crossing, the Cre-ER Egln1+/+ and Cre-ER Egln1fl/fl littermates were selected and used for further assays. A C57BL/6 mouse model with a point mutation (P10A) in the mouse Irf3 locus was generated by CRISPR/Cas9-mediated genome editing and obtained from the Cyagen Biosciences. Mice were housed (12-h light/dark cycle, 22°-26°C) and given unrestricted access to standard diet and tap water under specific pathogen-free conditions at the Animal Research Center of Wuhan University. The experimental mice were all 6-8 weeks old and the mice in the same experiment were the same age.<br>The egln1aihb1228/ihb1228 (egln1a−/−) (https://zfin.org/ZDB-ALT-180803-3), egln1bihb1229/ihb1229 (egln1b−/−) (https://zfin.org/ZDB-ALT-180803-4) and egln1aihb1228/ihb1228 egln1bihb1229/ihb1229(egln1a−/−egln1b−/−) double mutant zebrafish were previously described and used in this study. Zebrafish were bred and maintained in a recirculating water system according to standard protocols.
Wild animals	No wild animals used in this study.
Reporting on sex	The results of this study were not affected by the sex of the animals.
Field-collected samples	No field-collected samples used in this study.
Ethics oversight	The laboratory animal facility was accredited by the Association for Assessment and Accreditation of Laboratory Animal Care International (AAALAC), and all the animal procedures used in this study were approved by the Institutional Animal Care and Use Committee (IACUC) of the Institute of Hydrobiology, Chinese Academy of Sciences.

Note that full information on the approval of the study protocol must also be provided in the manuscript.

Flow Cytometry

Plots

Confirm that:

- [x] The axis labels state the marker and fluorochrome used (e.g. CD4-FITC).
- [x] The axis scales are clearly visible. Include numbers along axes only for bottom left plot of group (a 'group' is an analysis of identical markers).
- [x] All plots are contour plots with outliers or pseudocolor plots.
- [x] A numerical value for number of cells or percentage (with statistics) is provided.

Methodology

Sample preparation	The 6 weeks old Irf3_WT and Irf3_P10A mice were sacrificed, and lymphoid cells from spleen and intestinal lymph nodes were incubated with fluorochrome conjugated antibodies.
Instrument	BECKMAN COULTER CytoFLEX
Software	CytExpert
Cell population abundance	Expressed as the frequency of the selected population.

Gating strategy	Forward vs Side Scatter (FSC vs SSC) gating is first used to remove debris. Next, two parameter density plots by indicated marker are used as the subsetting gating.

☒ Tick this box to confirm that a figure exemplifying the gating strategy is provided in the Supplementary Information.
